# Time-dependent alterations in the rat nigrostriatal system after intrastriatal injection of fibrils formed by α–Syn and tau fragments

**DOI:** 10.3389/fnagi.2022.1049418

**Published:** 2022-11-28

**Authors:** Xiaoman Yang, Jialing Wang, Weiqi Zeng, Xiaoqian Zhang, Xiaomei Yang, Yu Xu, Yan Xu, Xuebing Cao

**Affiliations:** Department of Neurology, Union Hospital, Tongji Medical College, Huazhong University of Science and Technology, Wuhan, China

**Keywords:** α-synN103, tauN368, synucleinopathy, axonal transport, neurodegeneration, Parkinson’s disease

## Abstract

**Introduction:**

Accurate demonstration of phosphorylated α-synuclein aggregation and propagation, progressive nigrostriatal degeneration and motor deficits will help further research on elucidating the mechanisms of Parkinson’s Disease. α-synucleinN103 and tauN368, cleaved by activated asparagine endopeptidase in Parkinson’s Disease, robustly interacted with each other and triggered endogenous α-synuclein accumulation in a strong manner. However, the detailed pathophysiological process caused by the complex remains to be established.

**Methods:**

In this study, rats were unilaterally inoculated with 15 or 30 μg of this complex or vehicle (phosphate buffered saline, PBS). Over a 6-month period post injection, we then investigated the abundance of pSyn inclusions, nigrostriatal degeneration, and changes in axonal transport proteins to identify the various dynamic pathological changes caused by pSyn aggregates in the nigrostriatal system.

**Results:**

As expected, rats displayed a dose-dependent increase in the amount of α-synuclein inclusions, and progressive dopaminergic neurodegeneration was observed throughout the study, reaching 30% at 6 months post injection. Impairments in anterograde axonal transport, followed by retrograde transport, were observed prior to neuron death, which was first discovered in the PFFs model.

**Discussion:**

The current results demonstrate the value of a novel rat model of Parkinson’s disease characterized by widespread, “seed”-initiated endogenous α-Syn pathology, impaired axonal transport, and a neurodegenerative cascade in the nigrostriatal system. Notably, the present study is the first to examine alterations in axonal transport proteins in a PFF model, providing an appropriate foundation for future research regarding the mechanisms leading to subsequent neurodegeneration. As this model recapitulates some essential features of Parkinson’s disease, it provides an important platform for further research on specific pathogenic mechanisms and pre-clinical evaluations of novel therapeutic strategies.

## Introduction

Parkinson’s disease (PD) is one of the most common neurodegenerative diseases. It is characterized by motor symptoms, such as static tremor, rigidity, bradykinesia, and postural gait disorder. The pathological features of PD include loss of dopaminergic neurons in the nigrostriatal pathway and cytoplasmic proteinaceous inclusions, called Lewy bodies (LB; Przedborski, 2017). LB pathology was originally found in axons before becoming evident in neuronal soma, called “Lewy neurites” (LN; Koch et al., 2015). LB and LN are composed mainly of aggregated α-synuclein (α-Syn), a protein normally found pre-synaptically (Iba et al., 2020) that misfolds when phosphorylated at serine 129 (pS129; Hirai et al., 2004). Abnormal accumulation of α-Syn triggers the death of dopaminergic neurons in PD (Yasuda et al., 2013). However, the precise molecular and cellular mechanisms underlying the pathological proteinaceous inclusions remain to be determined.

Accurate experimental models may help to elucidate the role of pathological α-Syn in the mechanisms of neurodegeneration in PD. As one of the classic neurotoxicant models, chronic systemic exposure to rotenone mimics many features of PD (Sherer et al., 2003). Whereas adoption of such models is limited given variability in animal sensitivity and investigators. While several recent studies have reported α-Syn accumulations in transgenic and viral vector mediated models (Gombash et al., 2013; Nuber et al., 2013), the neurotoxicity in these models results from an overload of α-Syn, which is hardly representative of idiopathic PD.

Exposure to α-Syn preformed fibers (PFFs) leads to endogenous phosphorylation, accumulation, and formation of inclusions *in vivo* and *in vitro* (Volpicelli-Daley et al., 2011; Duffy et al., 2018). Such inclusions are characterized by the presence of phosphorylated α-synuclein (pSyn) and ubiquitin (Ub), proteinase K resistance and are thioflavin-positive. These features are similar to those observed in LB and appear to extend beyond the injected regions to reach synaptically connected areas (Paumier et al., 2015). However, motor deficit, a crucial feature of PD, is not well manifested in these models. In recent studies, the α-Syn PFFs-treated animals only manifested behavioral differences 6 months post-injection (Paumier et al., 2015; Patterson et al., 2019), making it not conducive for further research. Hence, more toxic PFFs which could mimic PD features and lead to rapid pathological changes is needed to build an appropriate α-synucleinopathy model.

Recent studies have demonstrated that activated asparagine endopeptidase (AEP) is highly expressed in PD, with the ability to cleave human α-Syn at N103 and tau at N368 (Zhang et al., 2014, 2017). The cleaved proteins co-localized and highly elevated in PD cases, and their complex PFFs are more potent in triggering endogenous α-Syn aggregation and neuronal death than their full-length (FL) proteins in mice after colonic injection (Ahn et al., 2020). We believe that this complex might be a more suitable promotor than α-Syn FL PFFs. However, the detailed pathological process triggered by such complex PFFs was not clear.

In this study, we used an intrastriatal injection of PFFs in rats. Rats were chosen for more complicated behaviors. Moreover, they are more comparable with humans in their genetic make-up and pharmacokinetics than mice. Then, the complex PFFs of α-SynN103/tauN368 replaced FL α-Syn PFFs to better fit PD pathology in humans. Moreover, we also used two doses of the PFFs complex, as the optimal concentration is unknown for rats. We chose to inject into the striatum based on studies suggesting that the pathogenesis of PD starts at the presynaptic terminals, and the striatum receive abundant nerve projections (Koch et al., 2015), especially from the substantia nigra (SN). Ultimately, rats were unilaterally intrastriatal inoculated with 15 μg or 30 μg PFFs complex or PBS vehicle, and assessed over a 6-month period. We investigated the abundance of pSyn inclusions, nigrostriatal degeneration, and axonal transport proteins, aiming to identify various and dynamic pathogenic changes caused by pSyn aggregates in nigrostriatal system.

## Materials and methods

### Experimental design

**Figure 1:**
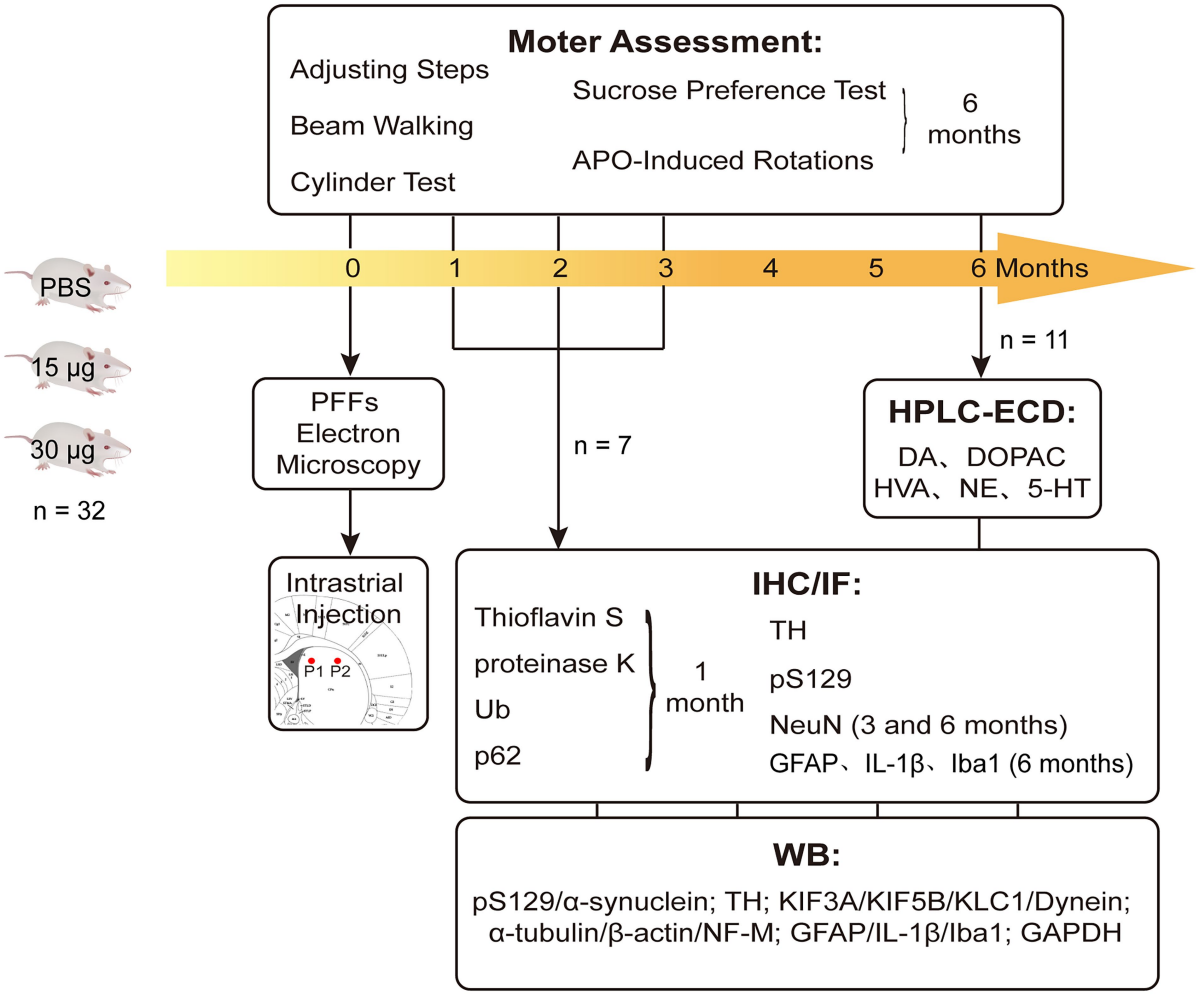
Experimental design and the endpoints. Rats were intra-striatal injected with either PBS vehicle or α-SynN103/tauN368 PFFs complex. Prior to injections, PFFs complex was tested with TEM to verify if effectively gathered. Locations of injections were pointed as P1 and P2 there. Brains were collected at 1, 2, 3 and 6 months post-injection for corresponding experiments. Behavioral tests were performed as well. IF measured thioflavin-S, α-Syn pS129, Ub and p62, as well as TH and NeuN. IHC measured α-Syn pS129, TH, NeuN and GFAP. WB was also performed to monitor nigrostriatal pathway changes, which contained α-Syn pS129, TH and axonal transport proteins.

Male, Sprague–Dawley (SD) rats (age: 6–7 weeks old; *n* = 96) were purchased from Vital River Laboratory Animal Technology Company (Beijing, China). They were housed in a specific pathogen free (SPF) animal laboratory with regulated temperature (21–23°C), a 12-h light/dark cycle, and *ad libitum* food and water. Our study was conducted in accordance with the Guidelines of Laboratory Animals Ethics of Huazhong University of Science and Technology. The proposal and research plan were approved by the Institutional Animal Care and Use Committee at Huazhong University of Science and Technology, China (IACUC number: 2592). The entire experimental flow diagram is shown in Figure 1.

### Generation of PFFs complex

Generation of PFFs were performed according to the protocol described by Ahn and colleagues (Ahn et al., 2020). The reaction system (500 μl per tube) containing 3 mg/ml of α-SynN103 and tauN368 protein monomers dissolved in PBS. The solution was incubated for 7 days at 37°C, with orbital shaking at 1000 rpms until the sample appeared cloudy. The PFFs were then validated by *in vitro* thioflavin S assay before injection.

### Transmission electron microscopy

Twenty microliters of the sample was placed on the carbon-coated copper grids and left for 3–5 min. Thereafter, 2% phosphotungstic acid was added onto the carbon-supported copper grids for 1–2 min; excess liquid after each step was soaked up with filter paper. The grids were allowed to dry at room temperature before imaging under a transmission electron microscope (HITACHI, HT7800/HT7700; Supplementary Figure S1).

### Stereotaxic injection

When they were at 7 weeks of age, rats were deeply anesthetized (pentobarbital sodium, 40 mg/kg weight) and fixed on a stereotaxic instrument. Next, an equal volume of PFFs (containing 15 μg or 30 μg PFFs complex) or PBS was injected into two sites of the right striatum using a 10-μl micro-syringe. The coordinates were as follows: anteroposterior (AP) +1.2 mm, mediolateral (ML) −2.4 mm, dorsoventral (DV) −4.0 mm; AP +0.2 mm, ML −3.5 mm, DV −4.0 mm. The animals were monitored with a temperature controller system until euthanized.

### Behavioral assessment

Behavioral analysis were applied prior to and at 1, 2, 3 and 6 months after intra-striatal injection, which included adjusting steps, beam walking and cylinder tests. Sucrose preference test and apomorphine (APO)-induced rotations were performed at 6 months.

#### Adjusting steps test

Rat’s hindquarters and contralateral forelimb were slightly lifted from the table, with only the testing forelimb touching the table. The number of steps taken by forelimbs in a straight forward distance of 90 cm within 5 s was counted, respectively, (Chen et al., 2017). The test was conducted three times for each rat, and the mean value was calculated and used for analysis to eradicate any discrepancies.

#### Beam walking test

The rats are expected to walk across an elevated narrow beam to a platform. The beam, with a cross section of 10 mm^2^ and a length of 1 m was placed at a height of 50 cm above the ground, and the end of the balance beam was connected to a non-transparent dark box. The test was conducted for three consecutive days: 2 days of training and 1 day of testing. On the day of the formal test, the time taken to pass the beam was recorded. The aforementioned conditions were based on modifications of tests on mice (Luong et al., 2011).

#### Cylinder test

The cylinder test measures the forelimbs use in a spontaneous exploration within a cylinder. Rats were individually placed in a transparent plexiglass cylinder (height, 16.5 cm; diameter, 24 cm) in a dimly lit room and observed for 5 min or until at least 20 forelimb movements were made. The numbers of unilateral and bilateral forelimbs use were recorded for each rat. Data are expressed as the percentage of contralateral forelimb use: [(left +1/2 both) divided by (left + right + both)] × 100, as previously described (Schallert, 2006).

#### Apomorphine-induced rotations test

To evaluate the extent of asymmetric striatal dopamine depletion, rats were assessed at 6 months after PFFs injection. Contralateral rotations were recorded within 30 min after subcutaneous apomorphine injection (0.05 mg/kg weight; Paillé et al., 2010). Data are expressed as the average number of rotations per minute.

#### Sucrose preference test

The test was performed to examine the effects of striatal lesions on depression in rats. Before the formal test, each rat underwent an adaptation training for a period of time. The rats were then evaluated as follows: an initial fasting period for 24 h, following which each rat was provided with one bottle of 1% sugar water and one bottle of plain water; the two bottles were swapped after 12 h (Avila et al., 2020). The volume of remaining water in both bottles was measured and converted to corresponding consumptions. The data are expressed as follows: % of sugar water preference = sugar water consumption/(sugar water consumption + plain water consumption) × 100%.

### Tissue preparation

After behavioral assessments, rats were sacrificed with isoflurane anesthesia. Subsequently, the brain tissue of four rats per group was rapidly removed on a pre-chilled plate and preserved at −80°C for western blotting. When we dissected the SN tissues, we did a ventral midbrain (VBM) dissection which mainly contained SN regions. The bilateral striatum of four other rats was removed for high-performance liquid chromatography (HPLC) at 6 months after injection. Moreover, three rats were transcranially perfused with 0.9% saline, followed by 4% ice-cold paraformaldehyde (PFA). Thereafter, their whole brains were completely removed and submerged in tissue fixative (4% PFA) for 48 h at 4°C. The fixed brain specimens were then dehydrated, embedded in paraffin, and sectioned for immunofluorescence and immunohistochemistry assays.

### Western blot analysis

Tissues were homogenized in RIPA lysis buffer (Servicebio, G2002) by ultrasonication for total protein extraction. The lysates then underwent centrifugation and measurement of protein concentrations using the BCA Protein Assay Kit (Boster, AR0146). Equal amounts of protein (20 μg) from each sample were separated on the SDS-PAGE gel and then transferred to a polyvinylidene difluoride membrane (Millipore, IPVH00010 and ISEQ00010), followed by blocking in 5% skimmed milk for 1 h at room temperature. After washing, the membranes were incubated overnight at 4°C with the following primary antibodies: anti-alpha-synuclein phospho S129 antibody (pS129, Abcam, ab51253), anti-tyrosine hydroxylase antibody (TH; Abcam, ab75875), anti-KLC1 antibody (Abcam, ab174273), anti-KIF3A antibody (Abcam, ab133587), anti-KIF5B antibody (Abcam, ab167429), anti-dynein antibody (Abcam, ab171964), anti-NF-M antibody (Proteintech, 25,805-1-AP), anti-α-tubulin antibody (Abbkine, A01080), anti-β-actin antibody (Abbkine, A01010), anti-GFAP antibody (Servicebio, GB12096), anti-IL-1β antibody (Proteintech, 16,806-1-AP), anti-α-synuclein (Abcam, ab212184) and anti-GAPDH antibody (Abbkine, A01020). All primary antibodies were diluted to a ratio of 1:1000. After incubation, the membranes were washed thrice and incubated for 1 h at room temperature with the appropriate secondary horseradish peroxidase (HRP)-conjugated antibody: goat anti-rabbit antibody (Abbkine, A21020) or goat anti-mouse antibody (Abbkine, A21010). After washing, an enhanced chemiluminescence kit (Biosharp, BL520A) was used to dye the membranes, and bands were detected through a gel imaging system (Syngene, United Kingdom). ImageJ software was used to analyze band intensities. The bands were firstly normalized through comparing with their GAPDH bands, and then compared among groups. The α-Syn bands served as endogenous α-Syn which remained unchanged, and α-Syn pS129 bands were normalized through comparing with α-Syn before reflecting as phosphorylated α-Syn triggered by the PFFs injection.

### Immunohistochemistry

Immunohistochemistry (IHC) was performed as previously described (Tan et al., 2020). Initially, brains were cut into 4-μm sections and mounted on slides. Thereafter, they underwent deparaffinization with xylene and rehydration with ethanol at graded concentrations; the sections were then water bathed in citrate solution (pH = 6.0) for antigen retrieval and washed with PBS (pH 7.4) trice. The sections were blocked in 3% bovine serum albumin (BSA) at room temperature and incubated overnight at 4°C with following primary antibodies: anti-TH antibody (Abcam, ab75875), anti-pS129 antibody (Biolegend, MMS-5091), anti-NeuN antibody (Proteintech, 26,975-1-AP), and anti-GFAP antibody (Servicebio, GB12096). All primary antibodies were diluted to a ratio of 1:1000. Thereafter, the sections were washed thrice and incubated with the appropriate secondary HRP-conjugated antibodies for 1 h at room temperature, followed by staining with 3,3′ -diaminobenzidine (DAB) solution for 5 min, counterstaining with Mayer’s hematoxylin (Absin, abs9215), and dehydration; eventually the slides were covered slipped. Images were collected using a digital slice scanning system (Olympus, VS120) and then analyzed using Image-J software. Sections containing the substantia nigra pars compacta (SNpc) were used for all counts. At least three areas of equal size (about 0.3 mm^2^) were drawn at the SNpc for each slide. The numbers of TH-positive and NeuN-positive cells in each box were manually counted, and the area fraction of pS129 were calculated using ImageJ after delineation of positive threshold. Results for the same animal were averaged for analysis.

### Proteinase-K digestion

Sections containing substantia nigra (SN) were washed with PBS thrice after water bath retrieval. They were then submerged in proteinase K solution (10 μg/ml, Biosharp, BS080) for 30 min at room temperature protected from light. Following digestion, the sections were then washed in PBS thrice and processed for IHC as described above.

### Immunofluorescence

Immunofluorescence (IF) staining was performed following the same procedure as IHC staining before secondary antibody incubation, with primary antibodies recognizing TH (1:1000, Abcam, ab75875), pS129 (1:1000, Abcam, ab51253), ubiquitin (1:500, Abcam, ab134953), and p62 (1:500, Abcam, ab109012), IL-1β (1:500, Proteintech, 16,806-1-AP). Thereafter, they were incubated for 50 min without light in related secondary antibodies, followed by incubation with 4′,6-diamidino-2-phenylindole (DAPI) solution for 10 min. Images were collected using a digital slice scanning system (Olympus, VS120). Sections containing the striatum were used for all counts. At least five areas of equal size were drawn at the striatum for each slide. The immunofluorescent density in each area was calculated using ImageJ after determining the positive threshold. Results for the same animal were averaged for analysis.

### Thioflavin S staining

Sections were deparaffinized and rehydrated as described above. They were then incubated with thioflavin S solution (0.3%, dissolved in 50% ethanol) at room temperature for 8 min, followed by incubation with DAPI solution as mentioned above. Images were collected using a digital slice scanning system (Olympus, VS120).

### High performance liquid chromatography coupled with electrochemical detection

Assessment of the levels of monoaminergic neurotransmitters was carried out as described previously (Zheng et al., 2018): 20 mg of tissues from the striatum was homogenized in ice-cold precipitation solution (0.1 M perchloric acid, 0.1% L-cysteine) by ultrasonication. The homogenates were then centrifuged and the supernatants were collected. Samples were separated on the Spherisorb ODS1 Column (5 μm, 4.6 mm × 250 mm, Waters, United States), and norepinephrine (NE), serotonin (5-HT), dopamine (DA), 3,4-dihydroxyphenylacetic acid (DOPAC) and homovanillic acid (HVA) were detected simultaneously. Compounds were detected using an electrochemical detector (HP, United States) under the following conditions: 30°C, detection potentials of 0.8 V, and a flow rate of 1 ml/min. The mobile phase was a mixture of HPLC grade methanol and buffer solution (3 mM sodium heptane sulfonate, 100 mM sodium acetate, 85 mM citric acid, and 0.2 mM EDTA) at a volume ratio of 92:8. Results were calculated and expressed as ng/mg wet weight.

### Statistics analysis

All data were analyzed using the GraphPad Prism 8.0 software and subjected to D’Agostino and Pearson test for normality. The comparisons between the two PFFs-treated groups regarding the area fraction of pS129 were performed using a Student’s *t*-test. One-way analysis of variance (ANOVA) was conducted for comparison among the three groups, followed by the Brown-Forsythe test and Tukey test for analyzing the differences between the two groups. Pearson’s correlation analysis was conducted between TH and NeuN positive counts. *p* values <0.05 were considered statistically significant.

## Results

### Intrastriatal injection of α–Syn N103/tau N368 complex PFFs initiates α–synucleinopathy in rats

**Figure 2:**
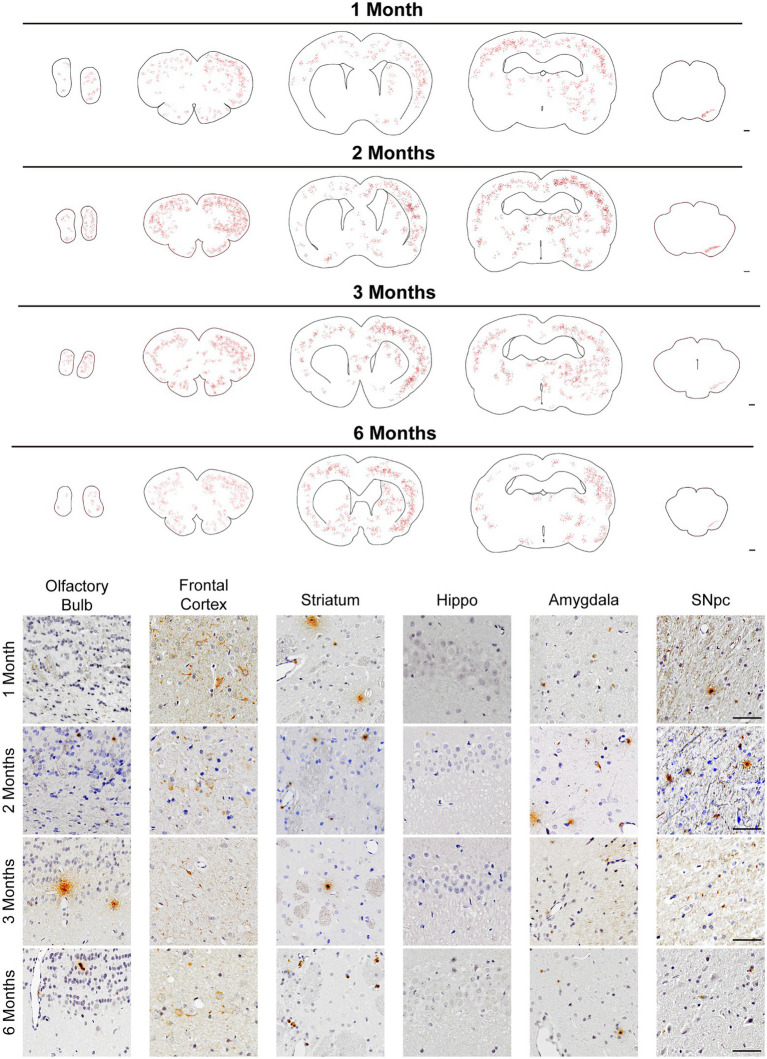
pSyn pathology spread throughout brain regions interconnected with the striatum. (Top) Schematics of representative sections throughout the rat brain, and inclusions were showed with red dots. Scale bar = 500 μm. pSyn aggregates spread to the anatomically connected brain regions including the SNpc, amygdala, cortex, and olfactory bulb. (Bottom) Micrographs from regions with prevalent pSyn aggregates at 1, 2, 3, and 6 months post-injection. Scale bar = 50 μm.

The dorsal striatum is interconnected with various cores in the central nervous system, including dopaminergic neurons in the midbrain (Volpicelli-Daley et al., 2011; Luk et al., 2012a). To figure out whether human original PFFs complex could initiate the pathological aggregations of endogenous α-Syn when injected into rats, we administrated IHC staining with anti-α-Syn pS129 antibody on a series of brain sections. Notably, we detected pronounced endogenous α-Syn pS129 pathology and spread to many regions connected to the striatum, including the olfactory bulb, frontal cortex, amygdala and SN pars compacta (SNpc), apart from the hippocampus (Figure 2). Even though we performed a unilateral injection, the pSyn was identified bilaterally in the frontal cortex, but remained unilaterally in the SNpc.

**Figure 3:**
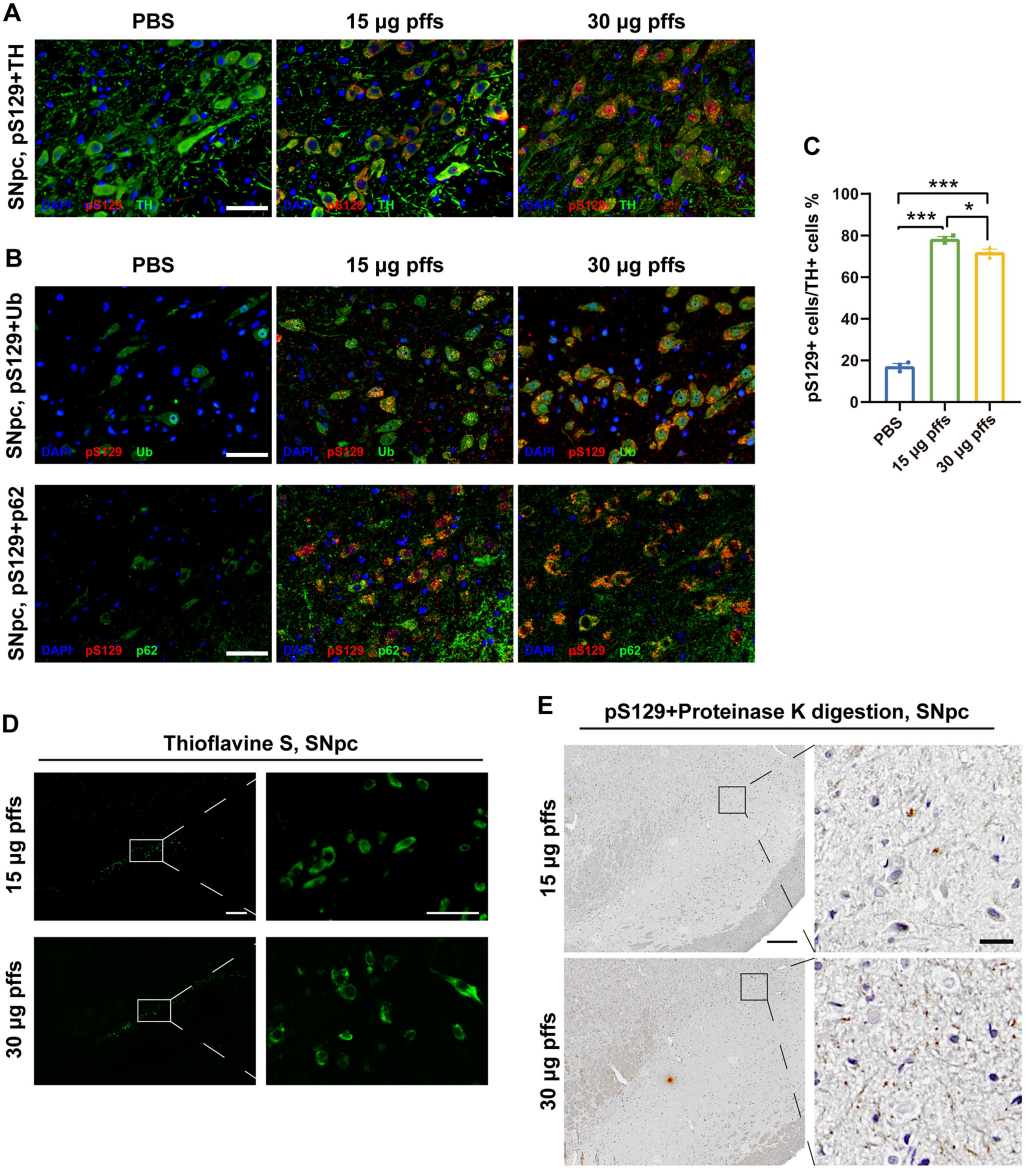
Intrastriatal injection of α-SynN103/tauN368 PFFs complex initiated α-synucleinopathy with common characteristics as human conditions in rats. **(A)** α-Syn pathology was detected in the ipsilateral SNpc. Scale bar = 50 μm. **(B)** α-Syn aggregates in the section of SNpc were confirmed by anti-α-Syn pS129, anti-p62 and anti-ubiquitin immunofluorescent staining. Scale bar = 50 μm. **(C)** α-Syn pS129-positive cells in the SNpc were quantified by counting (*n* = 3; *p < 0.05, ***p < 0.001). **(D)** Inclusions in the PFFs model were stained with thioflavin-S. High magnification images of boxed area showed on the right. Scale bar = 200 μm (left) and 50 μm (right). **(E)** Inclusions in the PFFs model were proteinase K resistant. High magnification images of boxed area were on the right. Scale bar = 200 μm (left) and 20 μm (right). All of the data was shown as means ± SEM.

To confirm that the pSyn inclusions were located in the dopaminergic neurons, we performed IF co-staining of pS129 and TH in SNpc (Figure 3A). Furthermore, the co-localization of pS129 with p62 and Ub indicated that they have common characteristics with LBs in the human condition (Figure 3B). In addition, significant thioflavin-S staining in the SNpc and proteinase-K resistant pSyn confirmed that the PFFs complex developed into insoluble aggregates (Figures 3D–E). Besides, we also conducted the IHC staining of α-Syn, which reflected the endogenous α-Syn and remained unchanged among groups (Supplementary Figures S2B–C).

Map of the phosphorylated α-Syn throughout the brain, from the olfactory bulb to the midbrain, demonstrated a time-dependent pattern of aggregation and propagation of LB-like inclusions (Figure 2 Top). Generally, pSyn aggregates initially increased in abundance over time, based on data from 1 to 2 months (Figure 2). However, aggregates constantly existed in the aforementioned regions, they became less abundant at 3 months in the SNpc and further decreased at 6 months. It is worth noting that pSyn aggregates developed much slower in the striatum, and it was not until 6 months post injection that this became evident. Within the 6 months, LBs-like pathology was not observed in regions that were not anatomically interconnected with striatum, such as the hippocampus.

In addition to assessing the α-Syn pathology after injection, we also conducted IHC staining of phospho-Tau (AT8). As predicted, AT8 activity was only detected in the injected sites (Supplementary Figure S2A), consistent with previous experiments (Ahn et al., 2020).

### Evaluation of the nigrostriatal system

#### Neuropathology at 1 month post striatal inoculation

**Figure 4:**
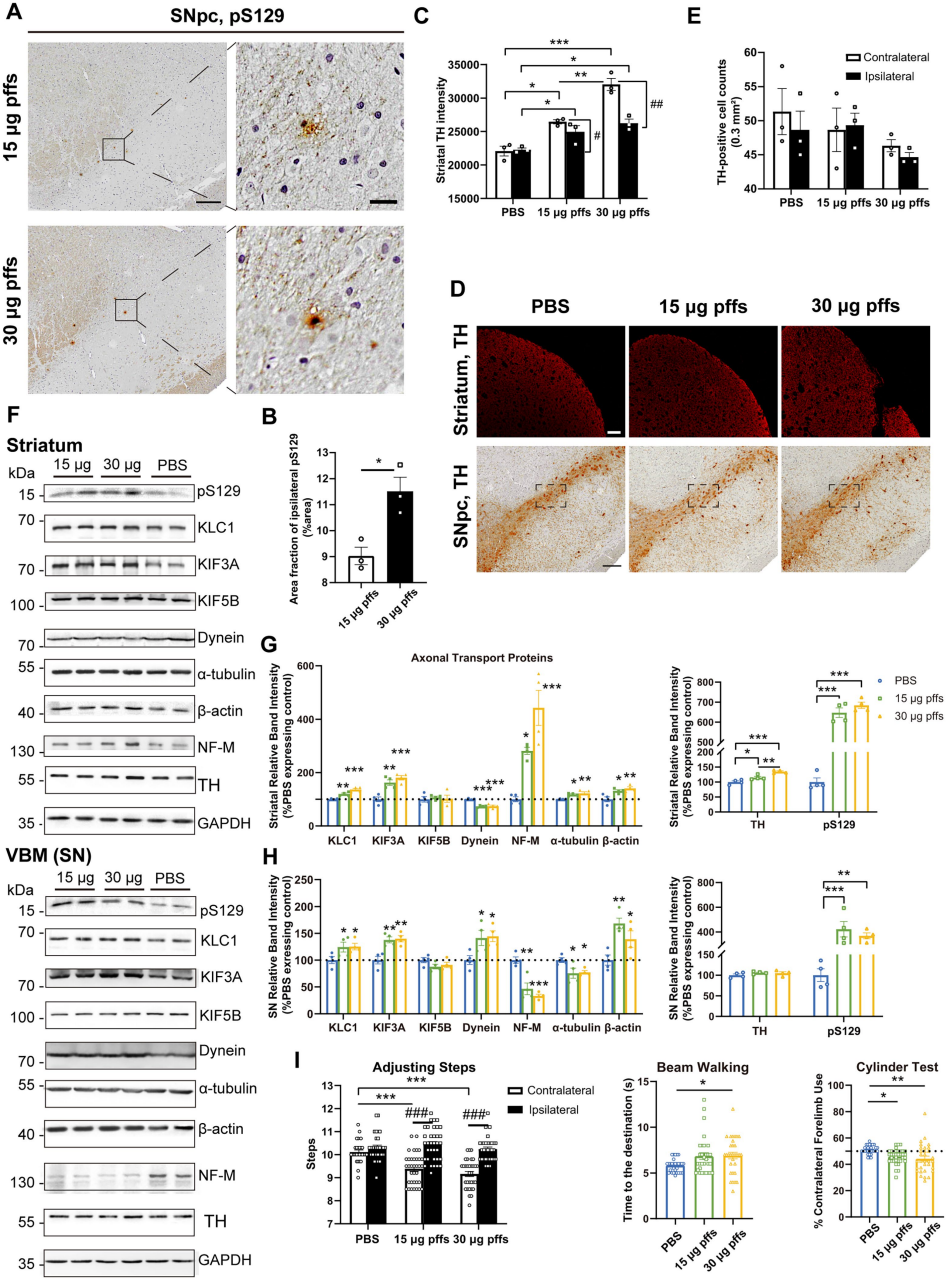
Evaluation of the nigrostriatal pathway at 1 month post injection. **(A)** α-Syn pathology was detected in the SNpc. Scale bar = 200 μm (left) and 20 μm (right). **(B)** The area fractions of the ipsilateral α-Syn pS129 in the SNpc of each group were quantified by ImageJ (*n* = 3; *p < 0.05). **(C)** Fluorescent densitometry of striatal TH (*n* = 3; *p < 0.05, **p < 0.01, ***p < 0.001; ^#^*p* < 0.05, ^##^*p* < 0.01). **(D)** TH-positive cells in the SNpc and striatum. Scale bar = 200 μm. **(E)** TH-positive cells in the SNpc were quantified by counting. The boxes represent the SNpc region (area = 0.3 mm^2^) where positive neurons were counted (*n* = 3). **(F)** Representative western blot of nigrostriatal changes. GAPDH served as the internal control. **(G,H)** Quantitative analysis of the afore-mentioned proteins. Data were presented as % of the PBS group (*p < 0.05, **p < 0.01, ***p < 0.001, *n* = 4). **(I)** Evaluation of behavioral changes (*n* = 32; *p < 0.05, **p < 0.01, ***p < 0.001; ^###^*p* < 0.001). All of the data was shown as means ± SEM.

At 1-month post injection, aggregates were hardly observed in the PBS controls or in the contralateral SNpc. In the ipsilateral SNpc, the area exhibiting pS129 positivity represented 9.03% ± 0.34% of the total area in the 15 μg group, and 11.51% ± 0.54% of the total area in the 30 μg group (Figures 4A,B).

Surprisingly, assessments of striatal denervation revealed a mild but significant increase in TH intensity compared with the PBS controls. Specifically, the ipsilateral striatum of the 30 μg group was near 120% intensity of the PBS control, and the 15 μg group was 110%. In the contralateral striatum, the 15 μg and 30 μg groups increased in TH intensity by 19.8 and 29.2%, respectively compared with the controls (Figures 4C,D).

With respect to the SNpc, however, no loss of TH-positive cells was observed in either of PFFs groups. Furthermore, neither the 15 μg nor the 30 μg group exhibited differences in the two hemispheres (Figures 4D,E).

An analysis of axonal transport protein expression revealed significant increases in the expression of the anterograde transport proteins KLC1 and KIF3A in the PFF-treated groups. Level of the retrograde transport protein, dynein was significantly decreased in the striatum but increased in the SN. As for the cytoskeletal proteins, levels of *β*-actin were significantly increased, whereas NF-M and α-tubulin levels increased in the striatum but decreased in the SN (Figures 4F–H).

#### Neuropathology at 2 months post striatal inoculation

**Figure 5:**
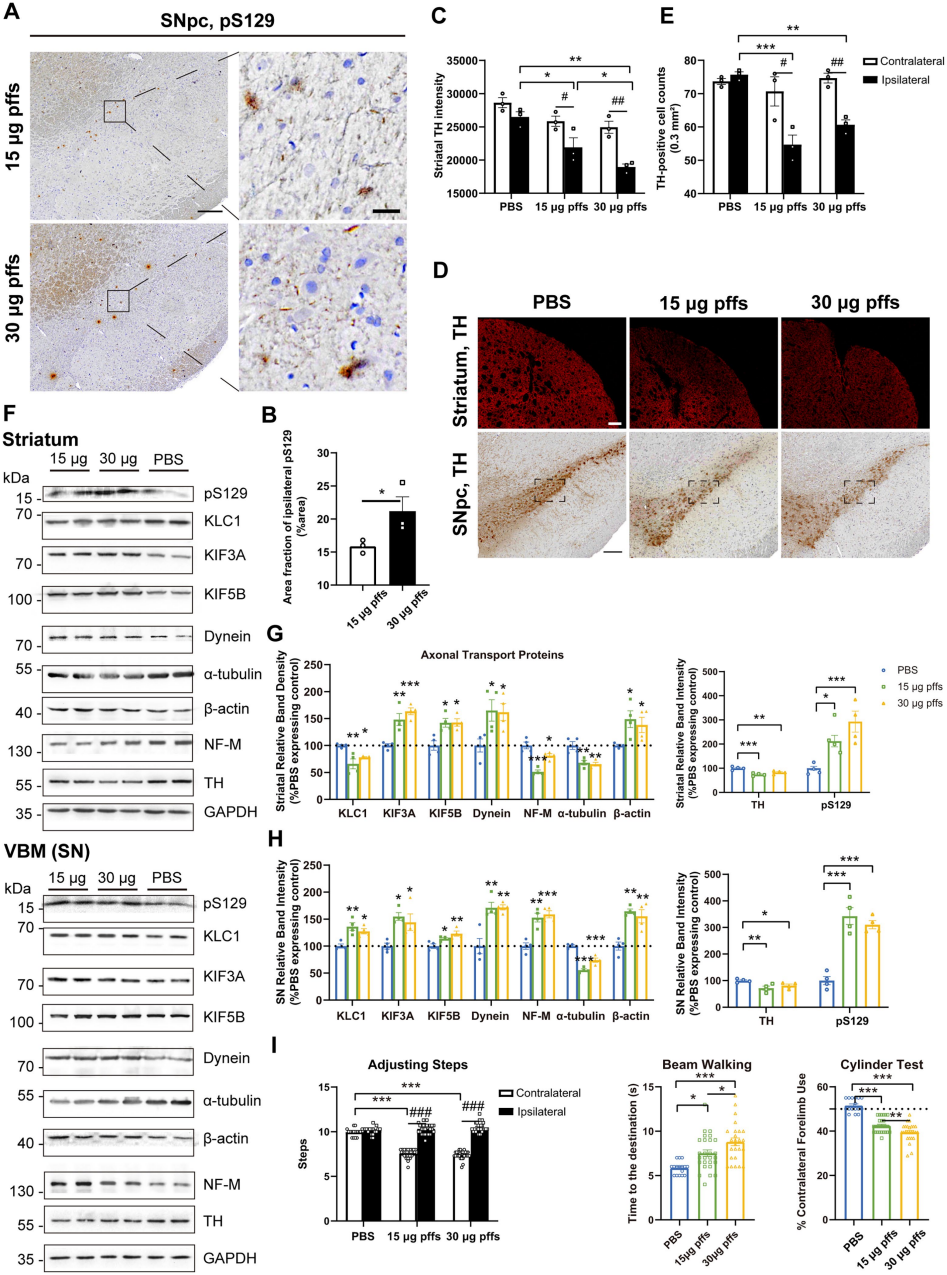
Evaluation of the nigrostriatal pathway at 2 months post injection. **(A)** α-Syn pS129 expression in the SNpc. Scale bar = 200 μm (left) and 20 μm (right). **(B)** The area fractions of the ipsilateral α-Syn pS129 were quantified by ImageJ (*n* = 3; *p < 0.05). **(C)** Fluorescent densitometry of striatal TH (*n* = 3; *p < 0.05, **p < 0.01; ^#^*p* < 0.05, ^##^*p* < 0.01). **(D)** TH-positive cells in the SNpc and striatum. Scale bar = 200 μm. **(E)** TH-positive cells in the SNpc were quantified by counting (*n* = 3; *p < 0.05, **p < 0.01, ***p < 0.001; ^#^*p* < 0.05, ^##^*p* < 0.01. **(F)** Representative western blot of nigrostriatal changes. **(G,H)** Quantitative analysis of the afore-mentioned proteins (*p < 0.05, **p < 0.01, ***p < 0.001; *n* = 4). **(I)** Evaluation of behavioral changes (*n* = 25; *p < 0.05, **p < 0.01, ***p < 0.001; ^###^*p* < 0.001). All of the data was shown as means ± SEM.

At 2 months post injection, we observed dose-dependent differences in pSyn inclusions in the ipsilateral SNpc between the two PFFs-treated groups (Figures 5A,B). Furthermore, the area fractions of pS129 reached 15.86 ± 0.57% in the 15 μg group and 21.17 ± 2.18% in the 30 μg group, representing increases of 76.0 and 83.9%, respectively, when compared with levels observed 1 month earlier.

We also observed a significant decrease in the ipsilateral striatum, without changes in the contralateral striatum (Figures 5C,D). Specifically, in the ipsilateral striatum, the TH intensity reduced to 82.8 and 70.9%, respectively compared with the PBS control.

In addition, we observed mild yet significant decreases in the numbers of TH-positive cells in the ipsilateral SNpc of PFFs groups, when compared with the controls and the contralateral hemisphere (Figures 5D,E).

Furthermore, PFFs complex induced further changes in axonal transport related proteins. On the one hand, KLC1 level, which had increased by 1 month, declined in the striatum. Levels of KIF3A and KIF5B were increased at this time. Increase in dynein level was also observed. Cytoskeletal proteins were totally changed. Expression of α-tubulin and β-actin remained downregulated and upregulated respectively, whereas NF-M expression increased in the SN but decreased in the striatum (Figures 5F–H).

#### Neuropathology at 3 months post striatal inoculation

**Figure 6:**
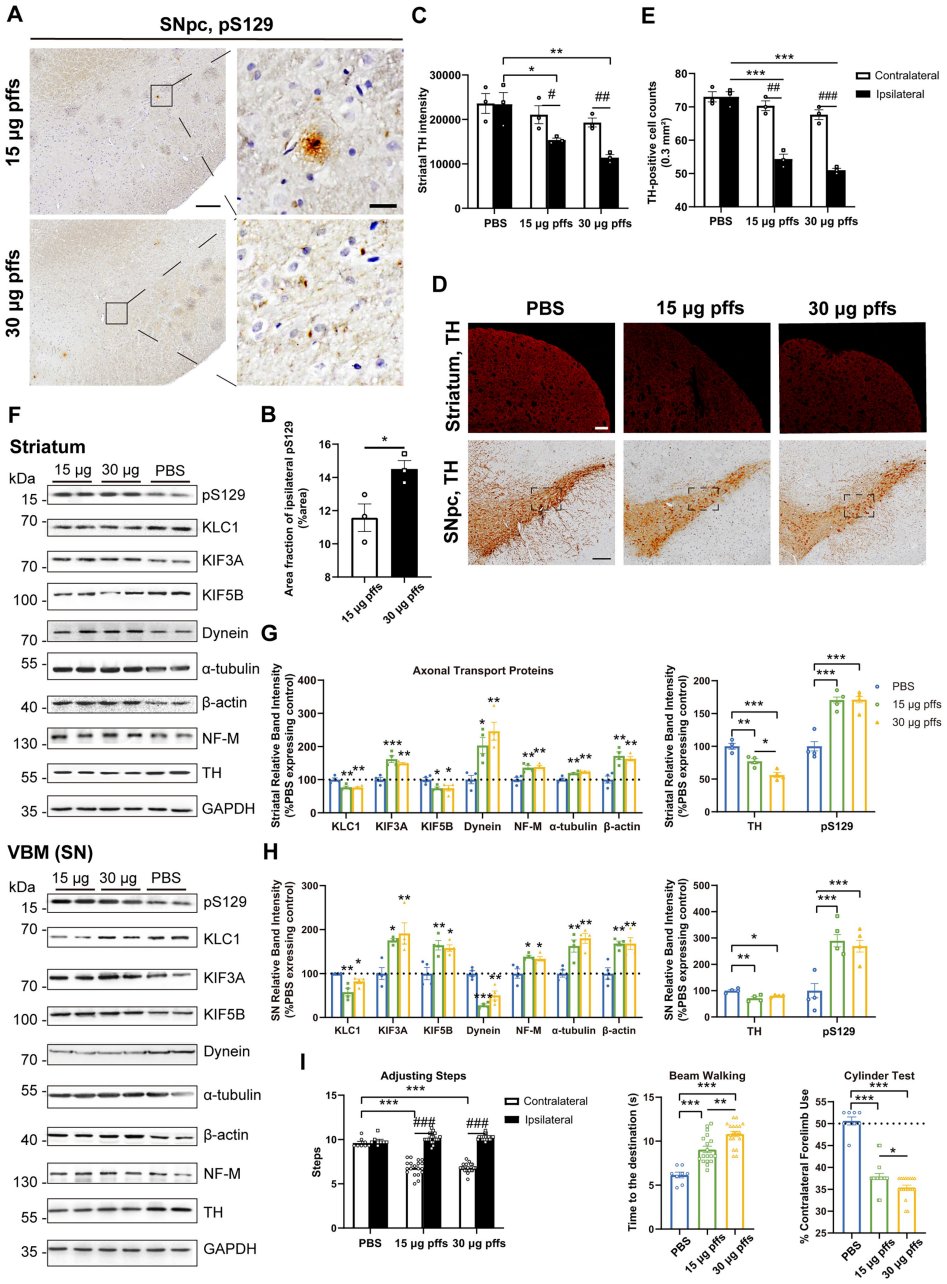
Evaluation of the nigrostriatal pathway at 3 months post injection. **(A)** α-Syn pS129 expression in the SNpc. Scale bar = 200 μm (left) and 20 μm (right). **(B)** The area fractions of ipsilateral α-Syn pS129 were quantified by ImageJ (*n* = 3; *p < 0.05). **(C)** Fluorescent densitometry of striatal TH (*n* = 3; *p < 0.05, **p < 0.01; ^#^*p* < 0.05, ^##^*p* < 0.01). **(D)** TH-positive cells in the nigrostriatal regions. Scale bar = 200 μm. **(E)** TH-positive cells in the SNpc were quantified by counting (*n* = 3; ***p < 0.001; ^##^*p* < 0.01, ^###^*p* < 0.001). **(F)** Representative western blot of nigrostriatal changes. **(G,H)** Quantitative analysis of the afore-mentioned proteins (*p < 0.05, **p < 0.01, ***p < 0.001; *n* = 4). **(I)** Evaluation of behavioral changes (*n* = 18; *p < 0.05, **p < 0.01, ***p < 0.001; ^###^*p* < 0.001). All of the data was shown as means ± SEM.

At 3 months post injection, the previously observed multiple pSyn aggregates were reduced. Specifically, the area fractions of pS129 had decreased by 27.0 and 31.5%, respectively, when compared with those observed at 2 months, as 15 μg group dropped to 11.57 ± 0.83% and 30 μg group to 14.50 ± 0.50%, with dose-dependent differences (Figures 6A,B).

TH immunoreactivity in the ipsilateral striatum was further reduced compared with the PBS control (Figures 6C,D). In particular, the TH intensity decreased to 65.6 and 48.7% in the 15 μg and 30 μg groups, respectively, compared with the PBS control.

The numbers of ipsilateral SNpc TH-positive cells became much lower in the PFF groups than those in the PBS group (Figures 6D,E). To investigate whether the loss of ipsilateral TH-positive neurons in the PFFs groups resulted from phenotypic or neuronal loss, cells within the SNpc were quantified using IHC with NeuN staining. There was no difference in NeuN-positive cells between the ipsilateral SNpc of PFFs groups and PBS control rats, suggesting a phenotypic down-regulation by 3 months (Supplementary Figures S3A,B).

Ultimately, the levels of axonal transport proteins changed considerably. Although KIF3A level kept increasing, KIF5B and KLC1 decreased. Meanwhile, dynein remained high in the striatum but deceased in the SN. Levels of all cytoskeletal proteins increased (Figures 6F–H).

#### Neuropathology at 6 months post striatal inoculation

**Figure 7:**
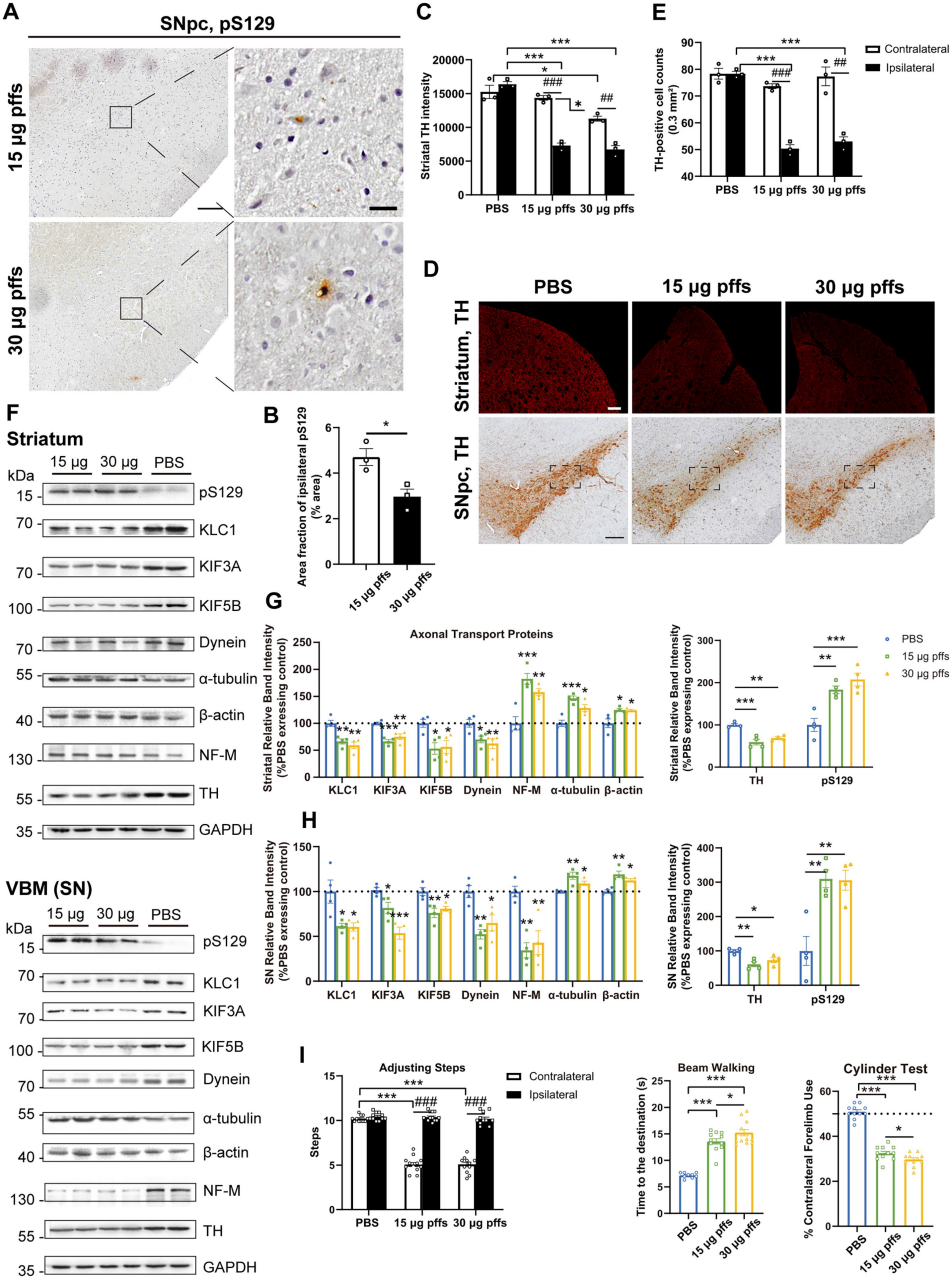
Evaluation of the nigrostriatal pathway at 6 months post injection. **(A)** α-Syn pS129 expression in the SNpc. Scale bar = 200 μm (left) and 20 μm (right). **(B)** The area fractions of the ipsilateral α-Syn pS129 in the SNpc were quantified by ImageJ (*n* = 3; *p < 0.05). **(C)** Fluorescent densitometry of striatal TH (*n* = 3; *p < 0.05, ***p < 0.001; ^##^*p* < 0.01, ^###^*p* < 0.001). **(D)** TH-positive cells in the nigrostriatal regions. Scale bar = 200 μm. **(E)** TH-positive cells in the SNpc were quantified by counting (*n* = 3; ***p < 0.001; ^##^*p* < 0.01, ^###^*p* < 0.001). **(F)** Representative western blot of nigrostriatal changes. **(G,H)** Quantitative analysis of the afore-mentioned proteins (*p < 0.05, **p < 0.01, ***p < 0.001; *n* = 4). **(I)** Assessment of behavioral changes (*n* = 11; *p < 0.05, ***p < 0.001; ^###^*p* < 0.001). All of the data was shown as means ± SEM.

At 6 months post injection, PFFs-treated groups displayed much fewer pSyn aggregates than those at earlier time points. Specifically, the area fractions of pS129 reduced to 4.70 ± 0.37% and 2.97 ± 0.33% in the 15 μg and 30 μg groups, respectively (Figures 7A,B).

Assessment of TH intensity in the striatum revealed a bilateral decrease in the 30 μg group compared with PBS controls (Figures 7C,D). Particularly, in the ipsilateral striatum, the 15 μg group exhibited a 55.42% loss and the 30 μg group exhibited a 58.88% loss of TH intensity compared with controls. Besides, in the contralateral striatum, the 30 μg group showed a mild but significant decrease.

Furthermore, quantification of TH-positive neurons in the SNpc revealed an ipsilateral decrease in both the 15 μg and 30 μg groups, compared with the PBS controls and the contralateral hemispheres (Figures 7D,E). Additionally, a decrease in NeuN-positive cells was observed in both the 15 μg and 30 μg PFFs groups compared with PBS group (Supplementary Figures S3D,E). The greater decrease in the number of the ipsilateral TH-positive cells was considered to reflect degeneration rather than a simple loss of TH phenotype (Supplementary Figure S3C).

Decreases in axonal transport protein expression and NF-M levels were observed in the SN, while the expression of other cytoskeletal proteins, α-tubulin and β-actin, dramatically increased (Figures 7F-H). To determine the possible reason for differential effects on motor and cytoskeletal proteins, we assessed astrocytes based on the levels of GFAP and IL-1β using western blotting. As expected, the expression of GFAP increased (Supplementary Figures S4B–E). Above results were confirmed by IHC staining (Supplementary Figures S4A,F). In alignment with that, the level of the proinflammatory cytokine, IL-1β, was significantly elevated (Supplementary Figures S4A–E,G). Collectively, these results suggest that nigrostriatal degeneration led to low levels of motor proteins, with augmented cytoskeletal proteins reflecting an activation of astrocytes.

### Behavioral assessments

To figure out whether intrastriatal PFFs injection cause any behavioral defects, various motor function assays were performed at each time point. Additionally, due to the greater decrease of nigral TH-positive cells and striatal dopaminergic innervations at 6 months, we supplied amphetamine-induced rotations and performed depression-like activity assays.

In the adjusting steps test, we observed mild but statistically significant differences between contralateral and ipsilateral forelimb use in PFFs treated groups at 1 month, and the differences gradually widened over subsequent months (Figures 4–7I left). Similarly, we also observed less contralateral forelimb use in cylinder test (Figures 4–7I right). Additionally, rats treated with PFFs complex took longer to arrive at the destination in the beam walking test (Figures 4–7I middle).

However, the PFFs-treated rats revealed no difference compared with PBS controls in rotations after amphetamine injection at 6 months.

**Figure 8:**
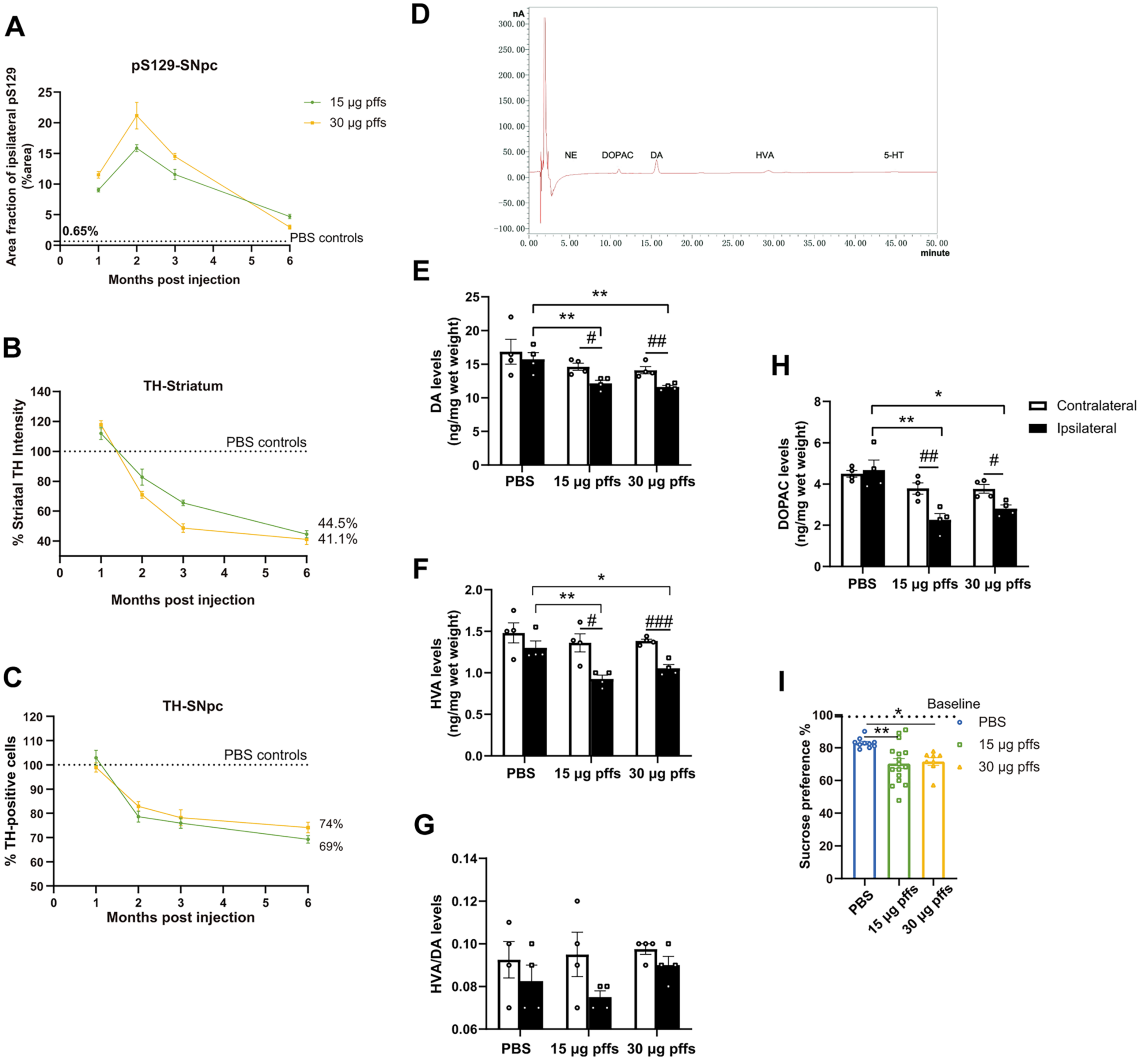
Temporal progression and features within the PFFs model. **(A)** Quantification of the pS129 in the ipsilateral SNpc. **(B,C)** Fluctuations of TH-positive cells in SNpc and striatum. Data were presented as % of the PBS group. **(D)** Sample chromatogram of 15 μg pffs solution. Peaks in order: NE (5 min), DOPAC (11 min), DA (16 min), HVA (30 min), 5-HT (45 min). **(E–H)** Increased α-Syn pathology lead to a loss of dopamine input to the striatum (*n* = 4; *p < 0.05, **p < 0.01; ^#^*p* < 0.05, ^##^*p* < 0.01, ^###^*p* < 0.001). **(I)** Sucrose preference test performed at 6-months post injection (*n* = 8–16; *p < 0.05, **p < 0.01). All of the data was shown as means ± SEM.

Moreover, the sucrose preference test was performed to assess depression-like activity, in which PFFs-treated rats showed less interest to sucrose compared with controls (Figure 8I).

### Longitudinal pathological changes associated with PFFs injections

Neuropathology owing to the intrastriatal injection of PFFs followed a time-dependent pattern and was affected by the quantity of injected complex (Figure 8A). Inclusions of pSyn in the ipsilateral SNpc peaked at 2 months and gradually decreased in the following months, which was more pronounced in the 30 μg PFFs group.

The ipsilateral striatum revealed dynamic alterations in TH intensity over the 6-month cohort (Figure 8B). TH immunoreactivity was upregulated within 1 month, followed by a gradual decrease over the next several months.

Ipsilateral loss of TH phenotype in SNpc was first significant at 2 months post treatment. The trend continued and resulted in further nigral neuron loss by 6 months (Figure 8C; Supplementary Figure S3C).

In alignment with the denervation of dopaminergic neurons in the striatum, unilateral injection of PFFs complex resulted in a 30% reduction in dopamine (DA; Figure 8C), and its metabolites, 3,4-dihydroxyphenylacetic acid (DOPAC) and homovanillic acid (HVA; Figures 8F,H). Furthermore, the HVA/DA ratio, which reflects dopamine turnover in CNS, was not significantly altered (Figure 8G). There was no significant difference among groups for striatal levels of norepinephrine (NE) and serotonin (5-HT).

## Discussion

Herein, we demonstrated that human α-SynN103/tauN368 complex PFFs successfully induced endogenous α-Syn phosphorylation, accumulation, and propagation after intrastriatal injection in rats. The α-synucleinopathy rat model fitted many features of sporadic PD, including progressive development of LB-like aggregates and loss of nigrostriatal dopaminergic neurons, in addition to axonal dysfunction before neuron death and significant neuroinflammation.

In the 6-month cohort, we observed temporal alterations in the nigrostriatal system. In particular, early compensatory upregulation of TH intensity in the bilateral striatum exceeded the loss at 1 month, and we observed no significant changes in TH of SNpc. This early event may reflect an attempt by the nigrostriatal system to maintain dopamine homeostasis. As reported, α-synN103 can bind and activate monoamine oxidase B (MAO-B). MAO-B catalyzes the oxidative deamination of monoamines and transforms DA into DOPAC (Zhang et al., 2017). As the rate-limiting enzymes in producing DA, TH expression may have upregulated to maintain striatal DA content, as previous studies demonstrated (Nakashima et al., 2013; Paumier et al., 2015; Patterson et al., 2019). At the second month, injection of PFFs resulted in an increased burden of inclusions and led to a significant downregulation of TH immunoreactivity in ipsilateral nigrostriatal system. The decrease of TH immunoreactivity within the striatum might reflect the striatal dopaminergic denervation, as the levels of neurofilaments (NF) reduced as well. However, it seems that the decrease in TH immunoreactivity in SNpc, though most of the PD-related researches regarded it as dopaminergic neuron death (Aras et al., 2014), represented more as phenotypic loss of TH rather than neuronal depletion, as NeuN-positive counts were unchanged until 6 months post injection. Consequently, previous results suggest that axonal degeneration precedes cell body changes, similar to early processes in PD (Kordower et al., 2013).

In our study, PFFs-treated rats exhibited endogenous α-Syn hyperphosphorylations and accumulations in several brain regions. The dissolvable aggregates were most prominent in the olfactory bulb, frontal cortex, amygdala, and SNpc. This implies that the pSyn aggregates tend to be present in multiple brain regions that innervate the injected striatum (Ahn et al., 2020). Even though pSyn aggregates spread bilaterally in the frontal cortex, they remained unilaterally in the SNpc, suggesting that the mechanism underlying the propagation is limited to synaptic connection (Wall et al., 2013). According to the pattern of pSyn aggregates observed, the PFFs complex seeded regressively from the injection site, as accumulations did not develop in striatal output regions, including the entopeduncular nucleus, globus pallidus and SN pars reticulata (Lee et al., 2020). Other regions, such as the hippocampus, which have functional connectivity with the striatum in memory-guided behaviors (Ferbinteanu, 2016), did not develop α-Syn pathology. This further confirmed that the spread of pSyn aggregates depends much more on anatomical connections, at least in the early stages. It was not until 6 months post injection that the contralateral striatum exhibited pSyn inclusions, unlike the contra-cortex. The frontal cortex projects bilaterally to the striatum, which contributes to the reverse transmission of α-Syn aggregates from the affected striatum to the contralateral cortex (Berendse et al., 1992). Hence, the presence of α-Syn pathology in the contra-striatum may represent a pathophysiological mechanism separated from the initial injection. This mechanism may involve release from the affected cells. The presence of pathologic α-Syn in the cerebrospinal fluid corroborates this mechanism (El-Agnaf et al., 2003). A recent study reported that microglia treated with α-Syn PFFs can release exosomes containing α-Syn, which then induce endogenous protein aggregation in multiple brain regions (Guo et al., 2020). Therefore, it is only a matter of time before the contralateral SN develops pSyn pathology, similar to clinical conditions in patients with unilateral nigrostriatal dysfunction that progresses over time to more severe bilateral nigrostriatal dysfunction (Marinus and van Hilten, 2015).

Furthermore, pSyn aggregates persisted in regions we observed initially throughout the 6-month cohort, and the abundance of the pathological accumulations gradually decreased from 3 months post injection. Additionally, the aggregated pSyn gradually shifted from neurites to cell bodies in the ipsilateral SNpc, as they transformed from thread-like to dense, rounded shapes, which were similar to LNs and LBs in PD (Volpicelli-Daley, 2017). The apparent decrease in pSyn aggregates may have been due to neurodegeneration. A previous report documented reduced dendritic spines within the cortex after intrastriatal injection of PFFs, suggesting that aggregates are cytotoxic and neurodegeneration could occur not only in the nigrostriatal system but also in other regions containing pathological inclusions (Blumenstock et al., 2017).

In clinical, Braak proposed a Braak pathological classification of PD (Braak et al., 2003), based on the different regions of abnormal α-Syn deposition in PD patients, and the nigrostriatal damage is associated with Stage 3. At this stage, Braak suggests that pathological α-Syn begins to accumulate in the midbrain, causing damage to the SNpc in particular. At the beginning of the stage, some mild yet asymmetric motor hypofunction appears several years before the typical clinical symptoms and can be called Pre-Clinical (Filippi et al., 2005), as presented by our models. With increasing age, the various factors, such as abnormal iron metabolism, reduced body mass, abnormal oxidative phosphorylation products *in vivo*, and telomere shortening, lead to increased deposition of Lewy Bodies in dopamine neurons (Vera et al., 2016; Deng et al., 2017; Cersosimo et al., 2018; Iglesias et al., 2018). In contrast, the animals used in our model were in middle age, when the above-mentioned pathogenic factors are not yet evident, which may be one of the reasons why α-Syn pathologies in PFFs-treated groups were not consistently increased in the later stages.

We observed more inclusions in the 30 μg group than in the 15 μg group in the first 3 months. However, this did not result in a greater damage, based on the TH phenotype in the nigrostriatal system. Moreover, the amounts of protein aggregates in the low-dose group surpassed those in the higher-dose group at 6 months. We hypothesized that richer pSyn aggregates led to a wider range of neuroinflammation; further, some of the pS129 we detected may have been located in activated astrocytes around the affected neurons, as we detected higher levels of GFAP and IL-1β in the 30 μg group than in the 15 μg group. Recent studies have demonstrated that suppression of astrocytic autophagy contributes to α-Syn inclusions (Lu et al., 2019). In other words, activated astrocytes might absorb and degrade pSyn aggregates to prevent further propagation and more extensive damage.

As tauN368, produced *via* the cleavage of tau by activated AEP, can induce tau phosphorylation (Zhang et al., 2014), we conducted IHC staining for AT8. Previous researches have demonstrated that both p-tau and α-Syn pathology can propagate over long distances within synaptically connected regions (Luk et al., 2012b; Sanders et al., 2014). However, we only observed α-Syn pathology in various brain regions. We did not observe tau phosphorylation in other regions apart from the injected striatum in rats. Moreover, a previous study noted that tau pathology can only spread in tau-overexpressed mice but not wild-type mice (Sanders et al., 2014; Ahn et al., 2020). Therefore, our findings indicate that transmission of tau pathology *in vivo* requires either α-Syn or Tau overexpression in transgenic mice. Hence, in our PFFs model, tauN368 was an inducer of α-Syn rather than tau phosphorylation and aggregation in neurons. As for the p-tau we observed in the ipsilateral striatum, hyperphosphorylation of tau can destabilize the microtubule network, which may have resulted in microtubule depolymerization (Iqbal et al., 2010), and interactions with α-Syn (Giasson et al., 2003), resulting in greater axonal degeneration (Yan et al., 2020).

Notably, the early formation of LNs emphasizes the importance of axonal pathology before neuronal death. Axonal transport plays a key role in several cellular processes, and alterations in levels of axonal transport proteins could reflect axonal pathology and contribute to the axonal degeneration associated with TH immunoreactivity in the striatum. With this in mind, we detected levels of proteins related to axonal transport (Table 1). At early time-points, several anterograde transport motor proteins were markedly upregulated in synaptic terminals, whereas retrograde transport proteins were reduced. In detail, kinesin and dynein exhibit a competitive relationship when transporting cargos (Steinberg, 2011), manifested as competition for overlapping microtubule-binding targets (Mizuno et al., 2004), and the “tug-of-war” confrontation against transport carriers (Belyy et al., 2016). The over-active anterograde axonal transport, in line with the increased TH phenotype, might reflect an attempt to maintain homeostasis of synaptic functions. Nevertheless, the attempt failed to control the pathological progress, as levels of KLC1 were decreased in the striatum, and the expression of dynein showed the opposite pattern. Such phenomenon may have resulted from a depletion in the anterograde axonal transport of certain cargoes and an upregulation of the retrograde axonal transport, leading to the accumulation of cargoes in the cell bodies. Later on, retrograde axonal transport was downregulated, which might influence the transport of extracellular signals and neurotrophic factor and result in somatic dysfunction (Volpicelli-Daley et al., 2014; Koch et al., 2015; Kang et al., 2017). Together, these findings indicate that, within the process of axonopathy, anterograde axonal transport is affected at first, followed by downregulation of retrograde axonal transport. However, the underlying molecular and cellular mechanisms remain to be established.

**Table 1:** Summary of the expression of axonal transport related proteins in the nigrostriatal system.

Region	Time point	Group	Indicators						
			KLC1	KIF3A	KIF5B	dynein	NF-M	α-tubulin	β-actin
Striatum	1 month	15 μg	↑↑	↑↑	ns	↓↓↓	↑	↑	↑
		30 μg	↑↑↑	↑↑↑	ns	↓↓↓	↑↑↑	↑↑	↑↑
	2 months	15 μg	↓↓	↑↑	↑	↑	↓↓↓	↓↓	↑
		30 μg	↓	↑↑↑	↑	↑	↓	↓↓	↑
	3 months	15 μg	↓↓	↑↑↑	↓	↑	↑↑	↑↑	↑↑
		30 μg	↓↓	↑↑	↓	↑↑	↑↑	↑↑	↑↑
	6 months	15 μg	↓↓	↓↓↓	↓	↓	↑↑↑	↑↑↑	↑
		30 μg	↓↓	↓↓	↓	↓↓	↑↑	↑	↑
SN	1 month	15 μg	↑	↑↑	ns	ns	↓↓	↓	↑↑
		30 μg	↑	↑↑	ns	↑	↓↓↓	↓	↑
	2 months	15 μg	↑↑	↑	↑	↑↑	↑↑	↓↓↓	↑↑
		30 μg	↑	↑	↑↑	↑↑	↑↑↑	↓↓↓	↑↑
	3 months	15 μg	↓↓	↑	↑↑	↓↓↓	↑	↑↑	↑↑
		30 μg	↓	↑↑	↑	↓↓	↑	↑↑	↑↑
	6 months	15 μg	↓	↓	↓↓	↓↓	↓↓	↑↑	↑↑
		30 μg	↓	↓↓↓	↓	↓	↓↓	↑	↑

↑ mild increase (*p* < 0.05), ↑↑ moderate increase (*p* < 0.01), ↑↑↑ significant increase (*p* < 0.001) in PFFs groups; ↓ mild decrease (*p* < 0.05), ↓↓ moderate decrease (*p* < 0.01), ↓↓↓ significant decrease (*p* < 0.001) in PFFs groups; ns, no significant difference.

As PD is characterized by motor deficits, the detection of motor behavioral impairments plays a critical role in PD models. As expected, rats treated with PFFs complex showed motor deficits as early as 1 month after injection in most behavioral tests, much earlier than observed in FL α-Syn PFFs models. Further, incoordination between the limbs increased over time, in parallel with the increase in striatal denervation from approximately 30% at 2 months to almost 60% at 6 months (Figure 8B). These results indicated that partial denervation can leads to mild yet quantifiable behavioral impairment (Boix et al., 2015).

Interestingly, the PFFs-treated rats manifested with depression-like behavior, measured by decreased sucrose preference. However, no changes in 5-HT or NE were detected in the striatum, which are established chemical correlates of depression. Recent researches have indicated that ventral tegmental area (VTA) dopamine neurons play a crucial role in susceptibility to social-stress-induced behavioral abnormalities (Douma and de Kloet, 2020). The inhibition of the VTA-medial prefrontal cortex projection can also promote susceptibility and manifest as decreased sucrose preference (Chaudhury et al., 2013). As the VTA develops pathological α-Syn in PFFs-treated mice (Ahn et al., 2020), we also conducted the co-staining of TH and pS129 there (Supplementary Figures S2D,E). As expected, the dopaminergic neurons developed phosphorylated aggregations and the TH intensity reduced compared to controls in the PFFs group. Hence, the anhedonia-like behavior observed in rats may be related to neurodegeneration of VTA dopamine neurons resulting from scattered α-Syn inclusions.

Despite the veracity with which our model approximated PD characteristics, several limitations of the model should be noted. First, early compensatory up regulation of TH expression in the striatum has not been discussed in clinical pathology of PD, and this may be even less relevant to the pathophysiology. In addition, we did not observe rotational behavior in the APO-induced rotation test. Asymmetric rotation is considered a classic behavioral feature of striatal DA depletion in hemi-lesioned animal models of PD, and has been correlated with a unilateral DA loss of more than 80% in the striatum (Miyanishi et al., 2019). Given that the depletion in PFFs-treated rats reached only 30%, our model may be more appropriate for approximating the early stages of PD.

In conclusion, we demonstrated a novel rat model of PD, characterized by “seed”-initiated widespread endogenous α-Syn pathology, impaired axonal transport and a neurodegenerative cascade in the nigrostriatal system. Notably, the present study is the first to examine alterations in axonal transport proteins in PFFs models, providing an appropriate foundation for future research regarding the mechanism leading to subsequent neurodegeneration. In accordance with significant dopaminergic denervation, unilateral striatal dopamine levels were also diminished in PFFs-injected rats, which demonstrated a disruption in motor coordination as early as 1 month post injection. As this model recapitulates some essential features of human conditions, it provides an important platform for further research on specific pathogenic mechanisms and the pre-clinical evaluation of novel therapeutic strategies.

## Data availability statement

The original contributions presented in the study are included in the article/Supplementary material, further inquiries can be directed to the corresponding authors.

## Ethics statement

The animal study was reviewed and approved by Institutional Animal Care and Use Committee at Huazhong University of Science and Technology, China.

## Author contributions

XiaomanY, JW, and XC contributed to the conception and design of the study. XiaomanY, JW, WZ, XiaomeiY, and YuX performed the experiments. XiaomanY and XZ performed the statistical analysis. XiaomanY and YanX wrote the first version of the manuscript. All authors contributed to the manuscript revision and approved the submitted version.

## Funding

This study was supported by grant from the National Natural Science Foundation of China (NSFC Project, No. 81873734 and 81974200).

## Conflict of interest

The authors declare that the research was conducted in the absence of any commercial or financial relationships that could be construed as a potential conflict of interest.

## Publisher’s note

All claims expressed in this article are solely those of the authors and do not necessarily represent those of their affiliated organizations, or those of the publisher, the editors and the reviewers. Any product that may be evaluated in this article, or claim that may be made by its manufacturer, is not guaranteed or endorsed by the publisher.
