# Supplementary material for: Time-dependent alterations in the rat nigrostriatal system after intrastriatal injection of fibrils formed by α–Syn and tau fragments
Source: Front Aging Neurosci. 2022 Nov 28;14:1049418. doi: 10.3389/fnagi.2022.1049418 (PMC9744116; doi:10.3389/fnagi.2022.1049418)

Supplementary Material

1 Supplementary Figures

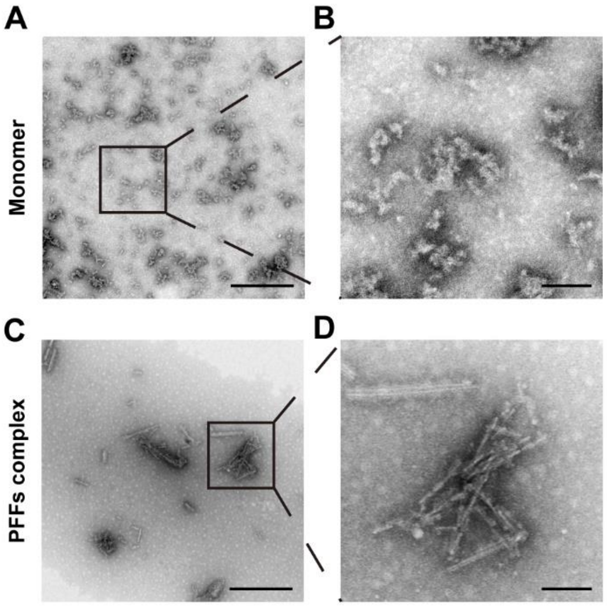

Supplementary Figure 1. Transmission electron micrographs. **(A)** Monomer and **(C)** preformed fibrils (PFFs) of α-SynN103/tauN368 complex. Scale bar = 500 nm. **(B, D)** Relative magnification. Scale bar = 100 nm.

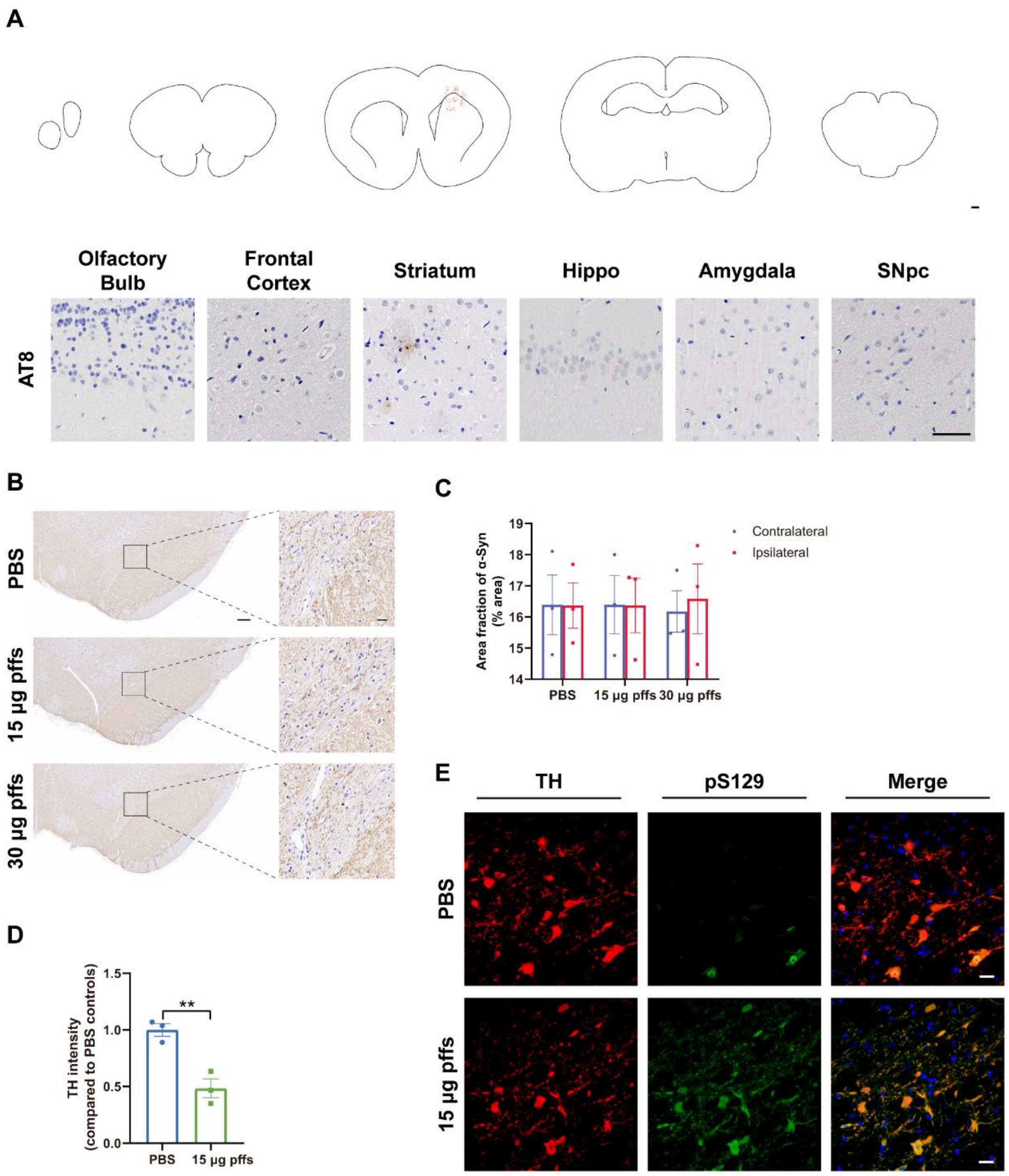

Supplementary Figure 2. α-SynN103/tauN368 PFFs injected in the striatum do not spread Tau pathology in rats. **(A Top)** Schematics of representative sections throughout the rat brain, and inclusions were showed with red dots. Scale bar = 500 μm. PFFs were intra-striatal injected, and brain sections were immune-stained by anti-AT8 antibody, after rats were sacrificed at 3 months. **(A Bottom)** The expression of AT8 was detected at only around the injected striatum, and did not spread to other brain regions. Scale bar = 50 μm. **(B)** IHC staining of α-Syn in SNpc. Scale bar = 200 μm (left) and 20 μm (right). **(C)** The area fractions of α-Syn were quantified by ImageJ, which remained no differences among groups. (n = 3 per group) **(D)** TH intensity in the VTA regions. (n = 3; **P < 0.01) **(E)** Co-staining of TH and pS129 in the VTA. Scale bar = 20 μm.

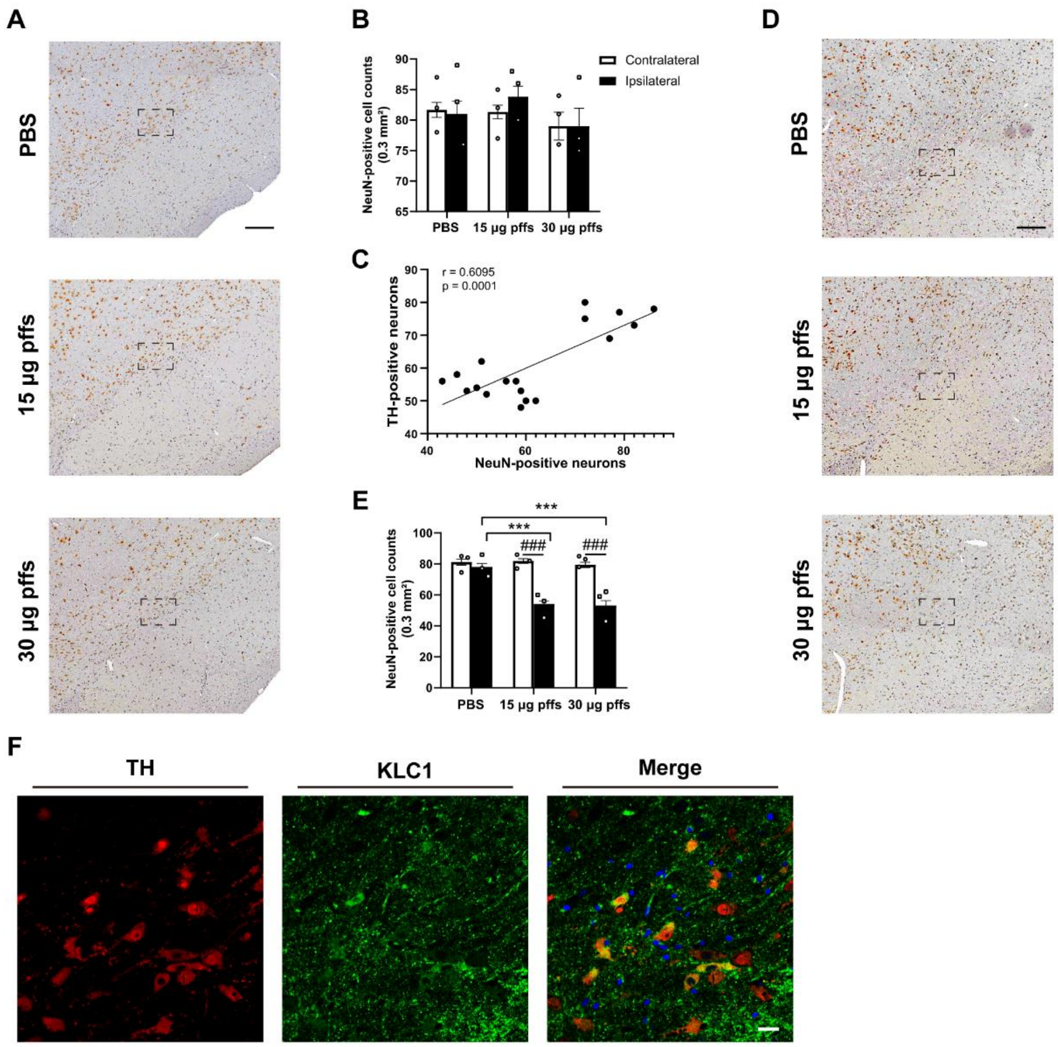

Supplementary Figure 3. Effects of PFFs injections on NeuN-positive cells at 3 and 6 months. NeuN-positive cells in the SNpc were analyzed by IHC staining. Ipsilateral SNpc of 3-months **(A)** and 6-months **(D)** were showed above. Scale bar = 200 μm. **(B, E)** NeuN-positive cells in SNpc of each group were quantified by counting. The boxes represent the region (area=0.3 mm^2) where positive neurons were counted. (n = 3; ***P<0.001; $^{###}$P < 0.001) **(C)** Pearson correlation plots for ipsilateral NeuN-positive and TH-positive neurons. All of the data was shown as means ± SEM. **(F)** Co-staining of TH and KLC1 in the SNpc. Scale bar = 20 μm.

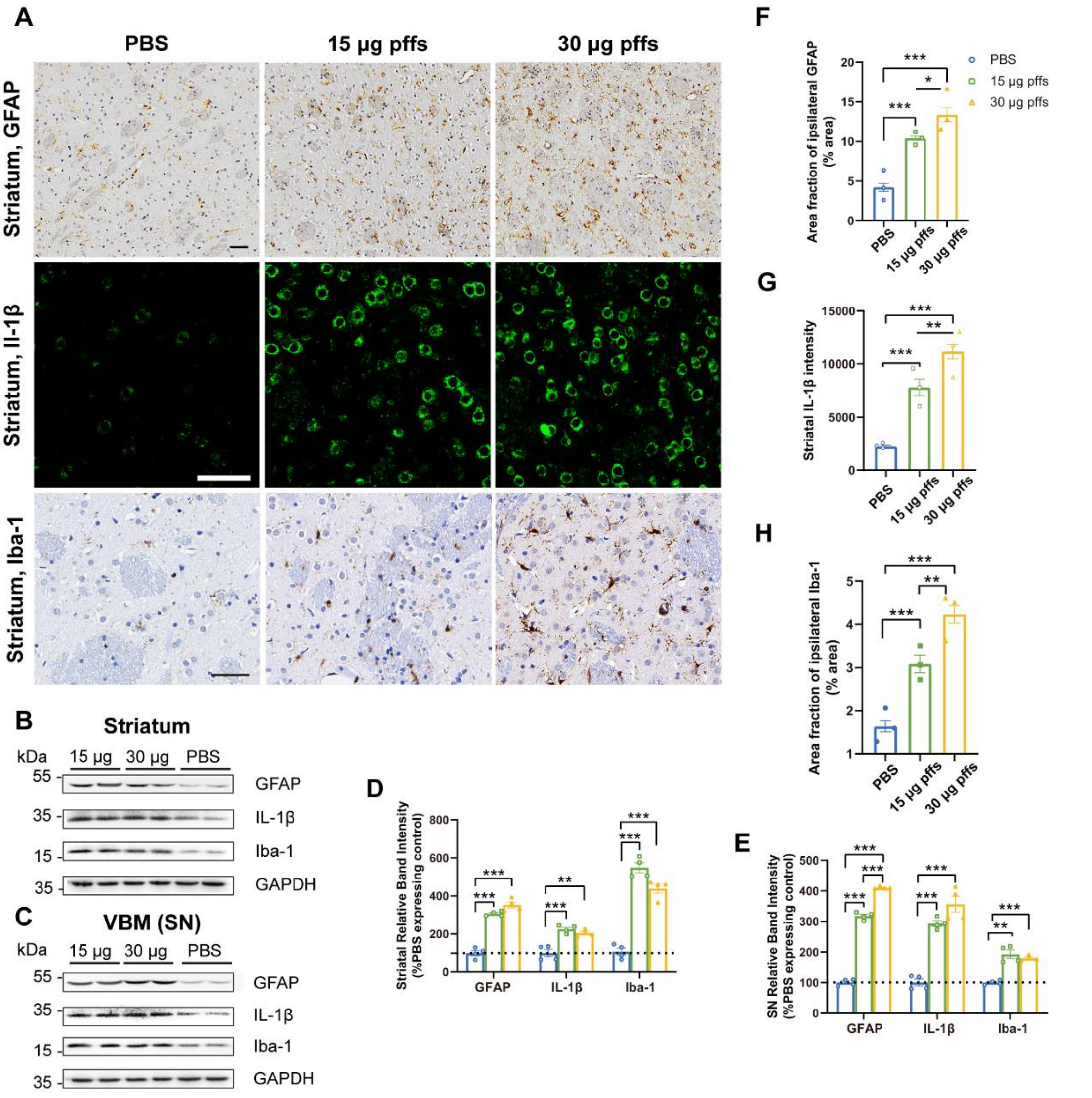

Supplementary Figure 4. Intrastriatal injection of α-synN103/tauN368 complex PFFs was associated with neuroinflammation. **(A)** Neuroinflammation was verified by IHC staining of GFAP **(top)** and Iba1 **(bottom)** and IF staining of IL-1β **(middle)**. Scale bar = 50 μm. **(B, C)** Representative western blot of nigrostriatal changes in the levels of GFAP, IL-1β and Iba1. GAPDH served as the internal control. **(D, E)** Quantitative analysis of the afore-mentioned proteins. Data were presented as % of the PBS group. (**P < 0.01, ***P < 0.001; n = 4) **(F, H)** The area fractions of the ipsilateral striatal GFAP **(F)** and Iba1 **(H)** were quantified by ImageJ. (n = 3; *P < 0.05, **P < 0.01, ***P < 0.001) **(G)** Fluorescent densitometry of striatal IL-1β. (n = 3; **P < 0.01, ***P<0.001) All of the data was shown as means ± SEM.

2 Supplementary Data

2.1 Full unedited blots for Figure 4:

2.1.1 Striatum:

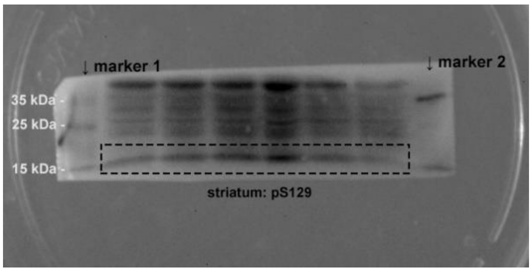
↓ marker 1
↓ marker 2
35 kDa -
25 kDa -
15 kDa -
striatum: pS129

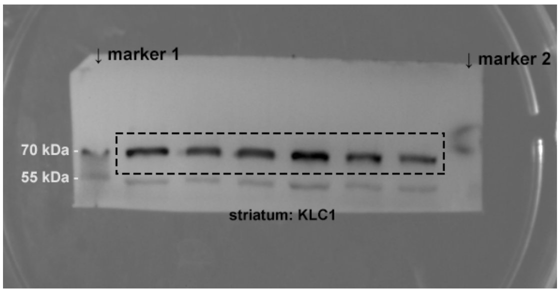
↓ marker 1
↓ marker 2
70 kDa -
55 kDa -
striatum: KLC1

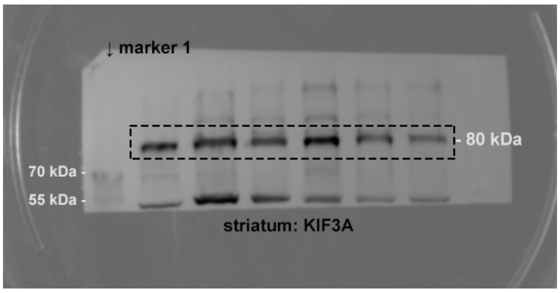
↓ marker 1
- 80 kDa
70 kDa -
55 kDa -
striatum: KIF3A

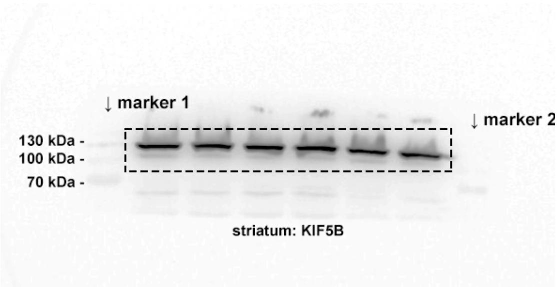
↓ marker 1
↓ marker 2
130 kDa -
100 kDa -
70 kDa -
striatum: KIF5B

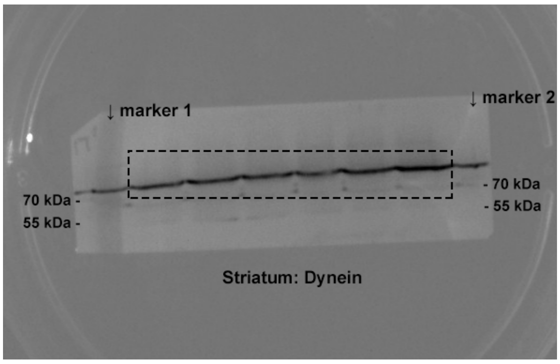
↓ marker 1
↓ marker 2
70 kDa -
55 kDa -
- 70 kDa
- 55 kDa
Striatum: Dynein

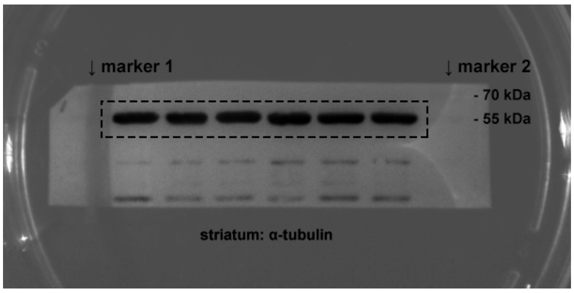
↓ marker 1
↓ marker 2
- 70 kDa
- 55 kDa
striatum: α-tubulin

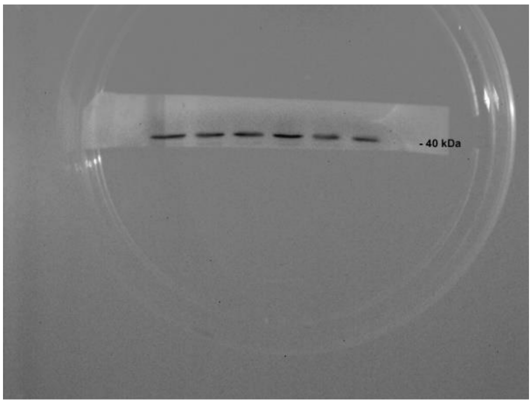
- 40 kDa

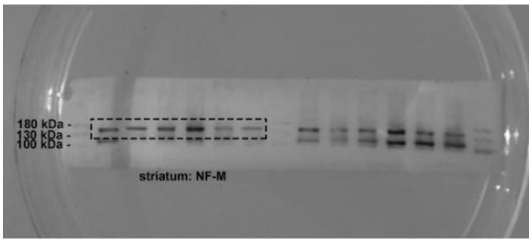
180 kDa -
130 kDa -
100 kDa -
striatum: NF-M

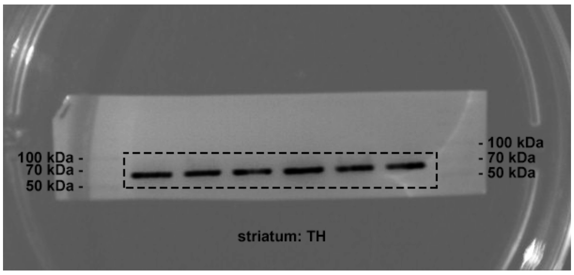
100 kDa -
70 kDa -
50 kDa -
- 100 kDa
- 70 kDa
- 50 kDa
striatum: TH

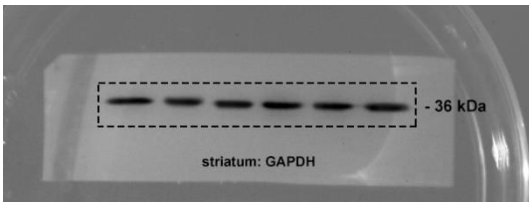
- 36 kDa
striatum: GAPDH

2.1.2 VBM (SN):

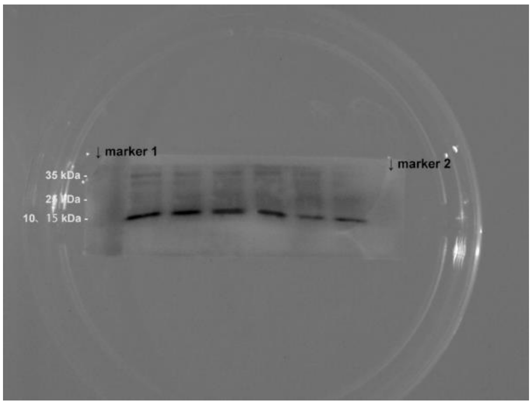
↓ marker 1
↓ marker 2
35 kDa -
25 kDa -
15 kDa -

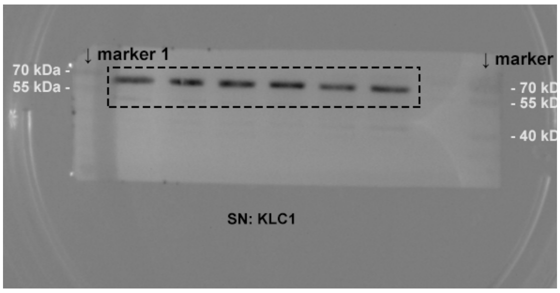
↓ marker 1
↓ marker
70 kDa -
55 kDa -
SN: KLC1

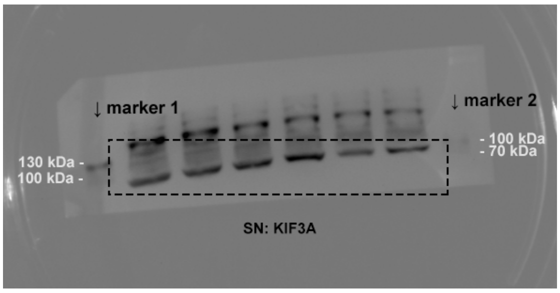
↓ marker 1
↓ marker 2
130 kDa -
100 kDa -
- 100 kDa
- 70 kDa
SN: KIF3A

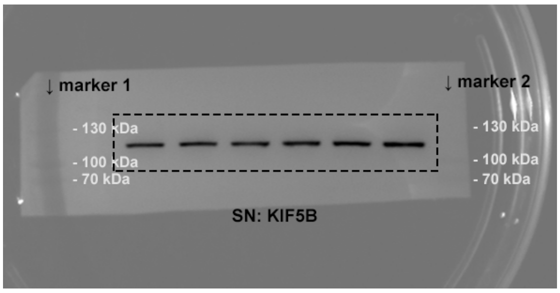
↓ marker 1
↓ marker 2
- 130 kDa
- 100 kDa
- 70 kDa
SN: KIF5B

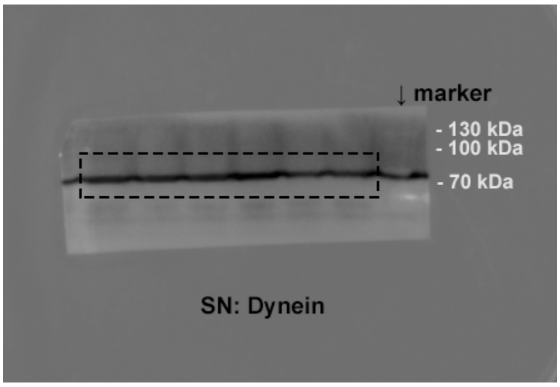
↓ marker
- 130 kDa
- 100 kDa
- 70 kDa
SN: Dynein

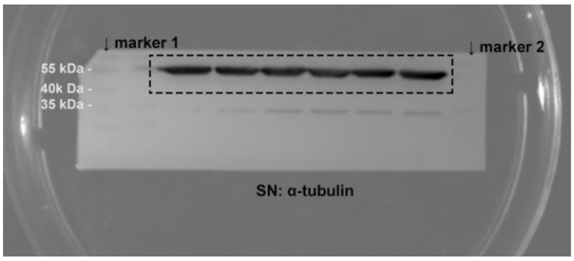
↓ marker 1
↓ marker 2
55 kDa -
40k Da -
35 kDa -
SN: α-tubulin

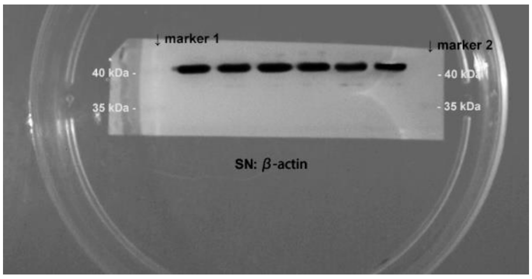
↓ marker 1
↓ marker 2
40 kDa -
35 kDa -
- 35 kDa
SN: β-actin

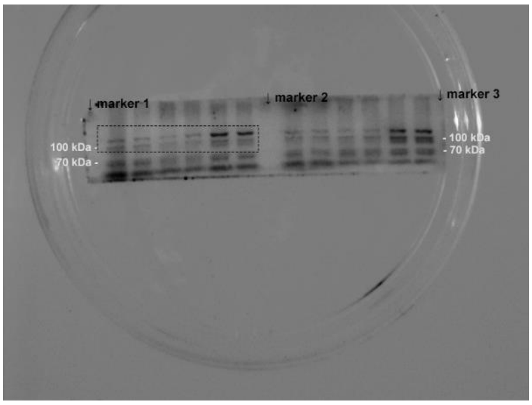
↓ marker 1
↓ marker 2
↓ marker 3
100 kDa -
70 kDa -
- 100 kDa
- 70 kDa

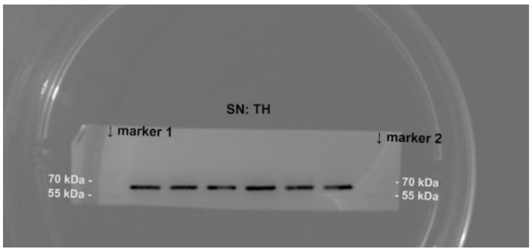
SN: TH
↓ marker 1
↓ marker 2
70 kDa -
55 kDa -
- 70 kDa
- 55 kDa

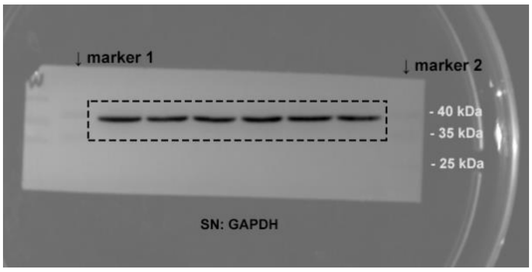
↓ marker 1
↓ marker 2
- 40 kDa
- 35 kDa
- 25 kDa
SN: GAPDH

2.2 Full unedited blots for Figure 5:

2.2.1 Striatum:

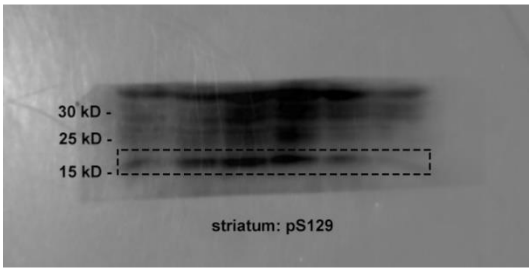

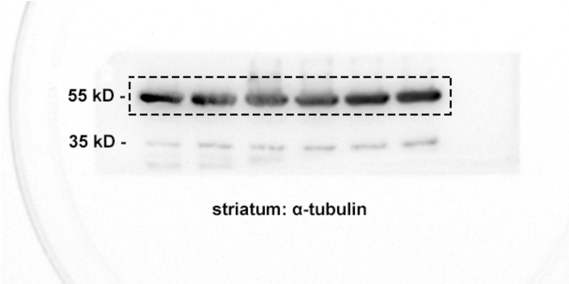

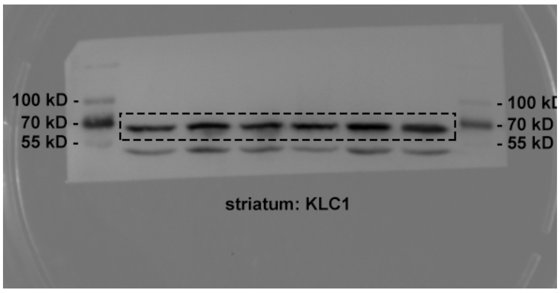

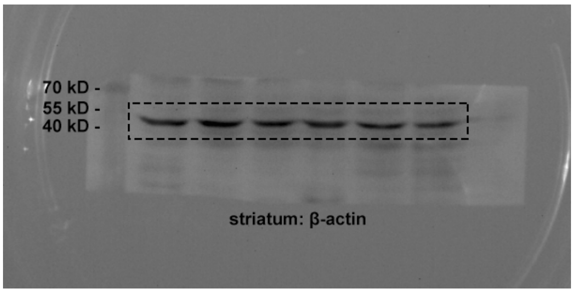

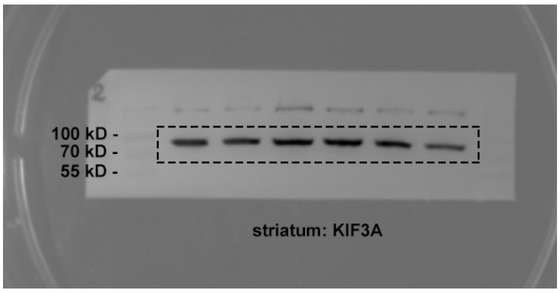

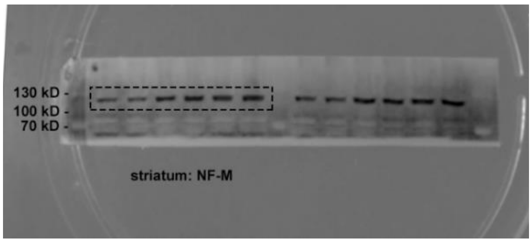

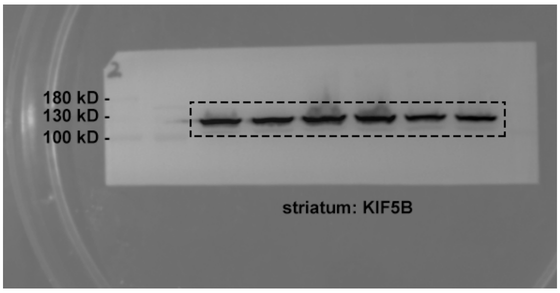

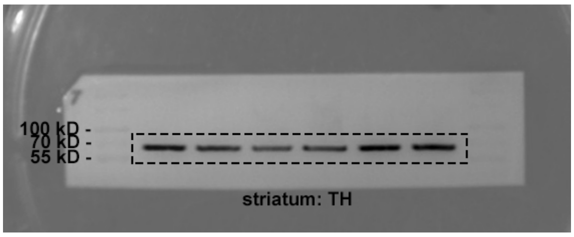

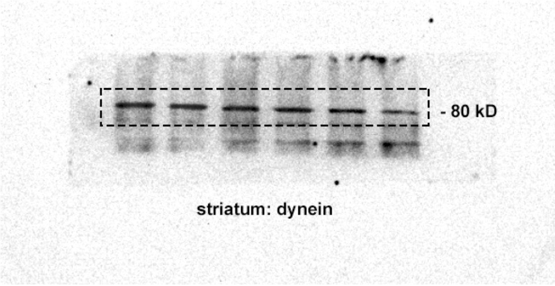

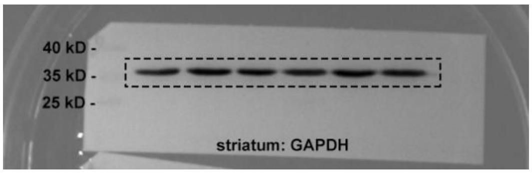

2.2.2 VBM (SN):

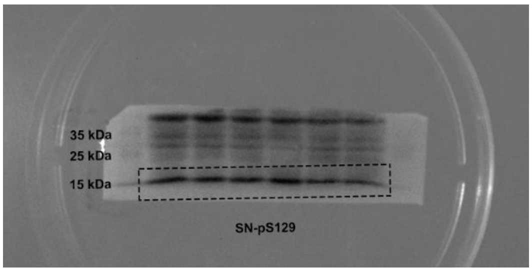
35 kDa
25 kDa
15 kDa
SN-pS129

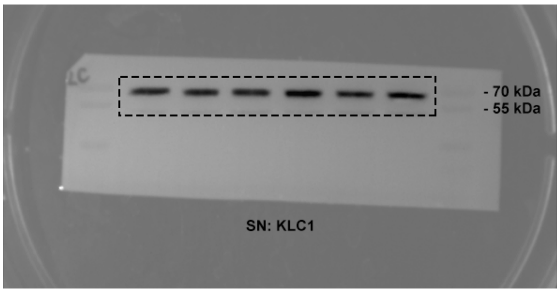
- 70 kDa
- 55 kDa
SN: KLC1

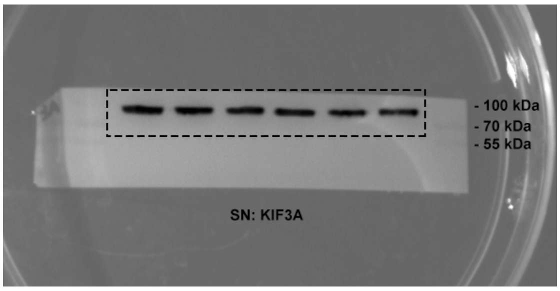
- 100 kDa
- 70 kDa
- 55 kDa
SN: KIF3A

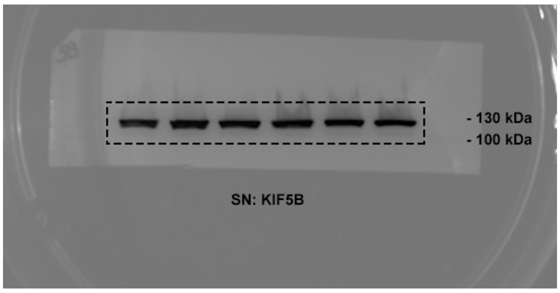
- 130 kDa
- 100 kDa
SN: KIF5B

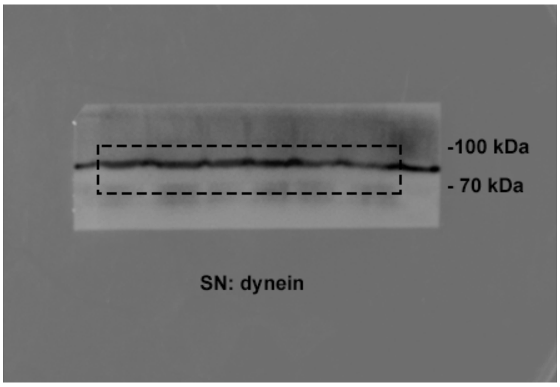
-100 kDa
- 70 kDa
SN: dynein

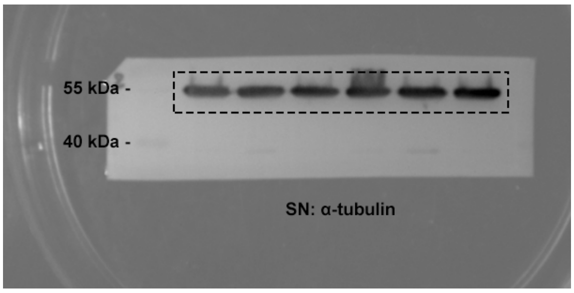
55 kDa -
40 kDa -
SN: α-tubulin

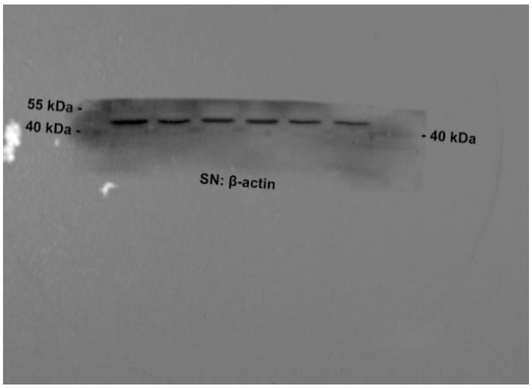
55 kDa -
40 kDa -
- 40 kDa
SN: β-actin

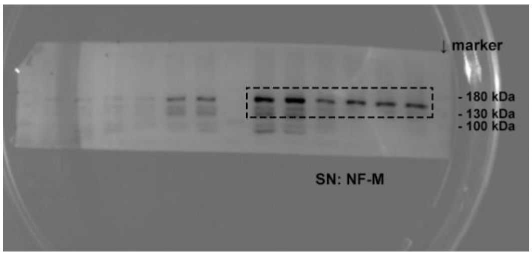
↓ marker
- 180 kDa
- 130 kDa
- 100 kDa
SN: NF-M

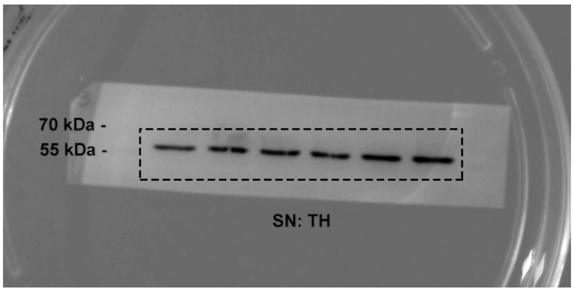
70 kDa -
55 kDa -
SN: TH

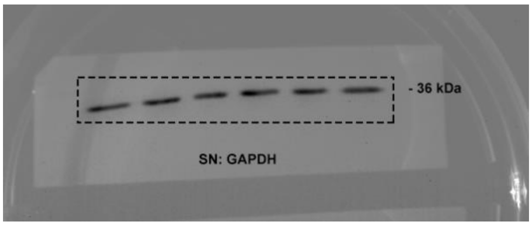
- 36 kDa
SN: GAPDH

2.3 Full unedited blots for Figure 6:

2.3.1 Striatum:

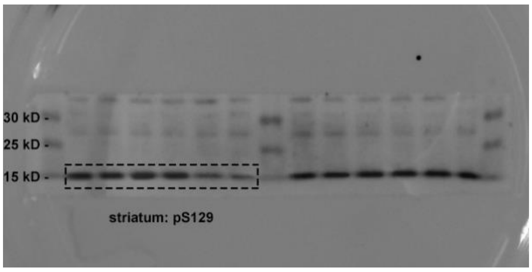

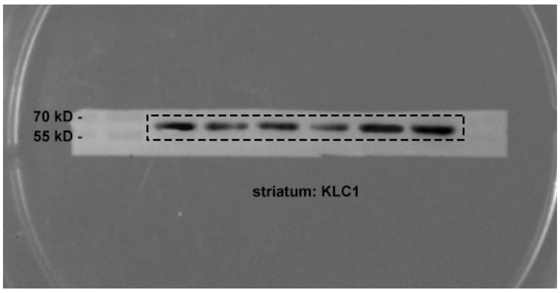

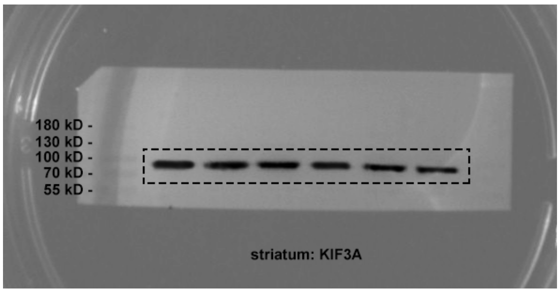

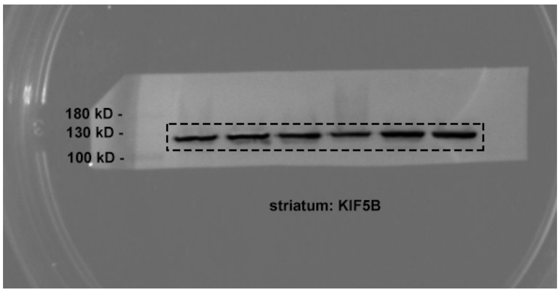

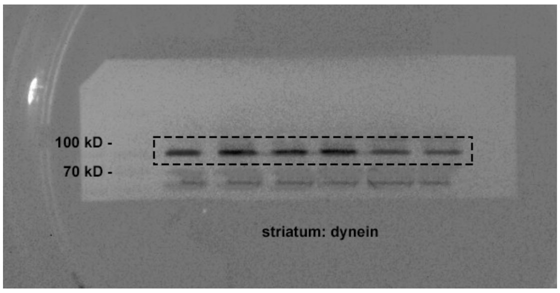

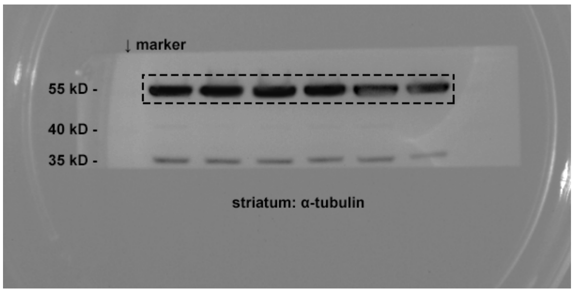

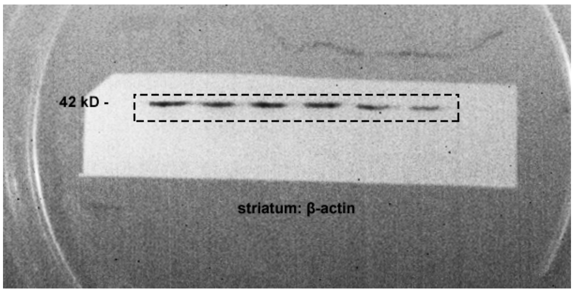

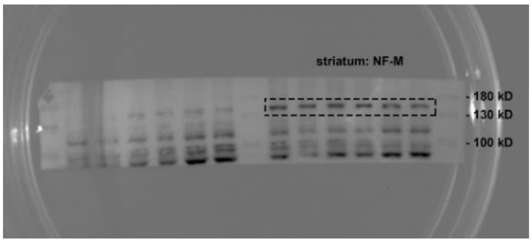

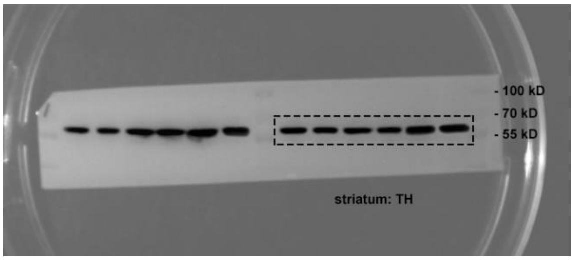

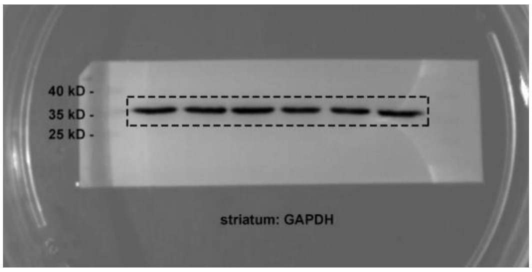

2.3.2 VBM (SN):

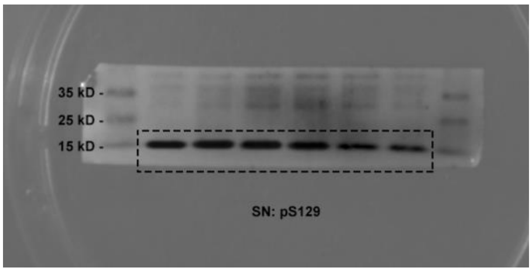
35 kD -
25 kD -
15 kD -
SN: pS129

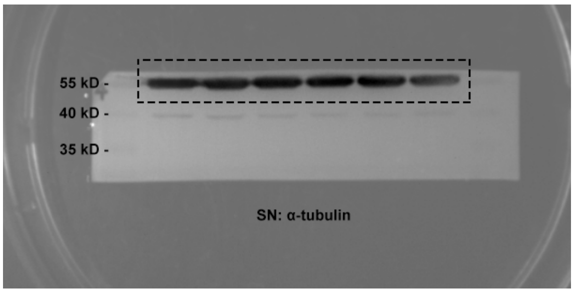
55 kD -
40 kD -
35 kD -
SN: α-tubulin

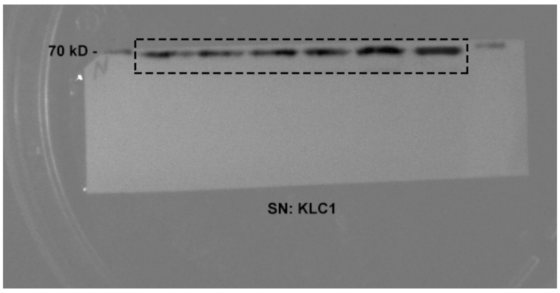
70 kD -
SN: KLC1

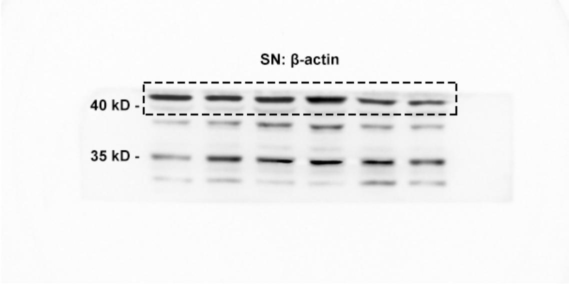
SN: β-actin
40 kD -
35 kD -

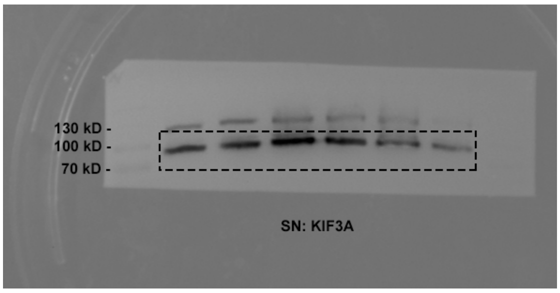
130 kD -
100 kD -
70 kD -
SN: KIF3A

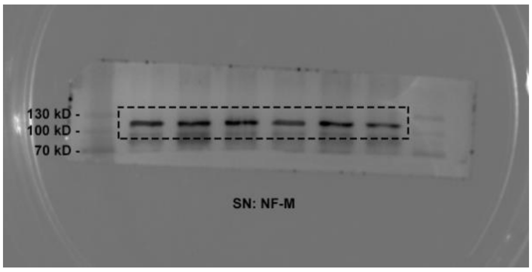
130 kD -
100 kD -
70 kD -
SN: NF-M

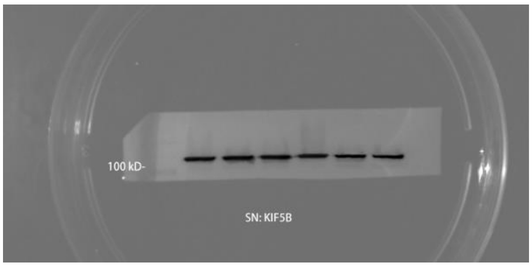
100 kD-
SN: KIF5B

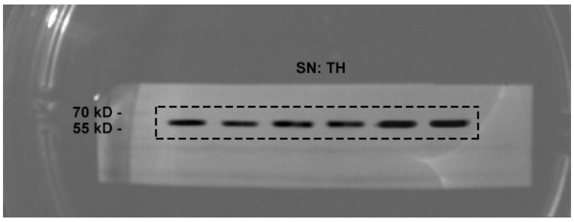
SN: TH
70 kD -
55 kD -

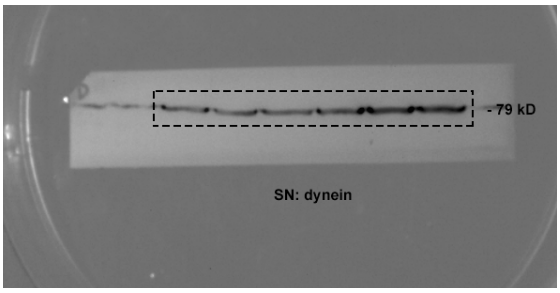
- 79 kD
SN: dynein

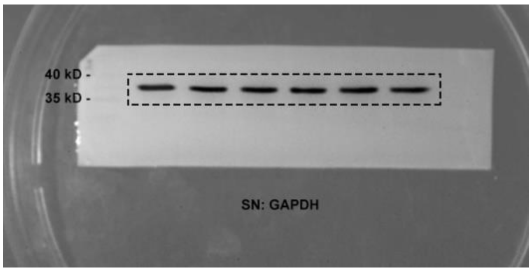
40 kD -
35 kD -
SN: GAPDH

2.4 Full unedited blots for Figure 7:

2.4.1 Striatum:

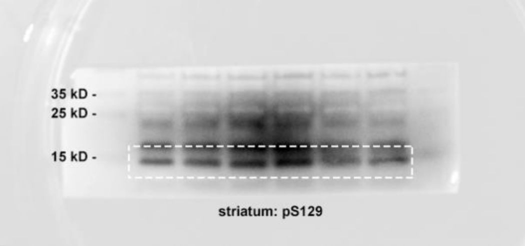

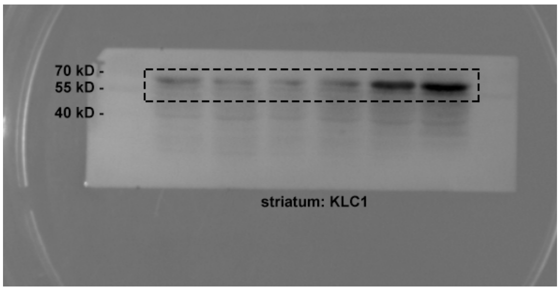

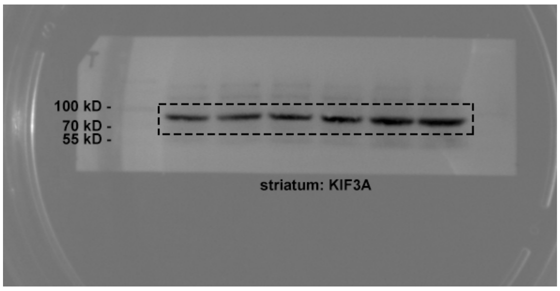

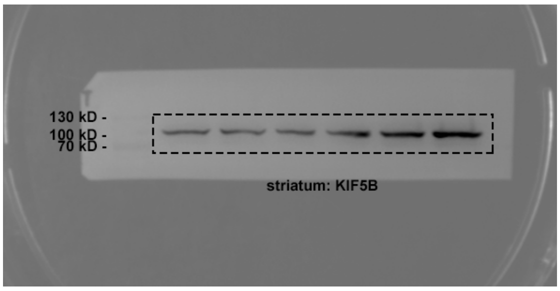

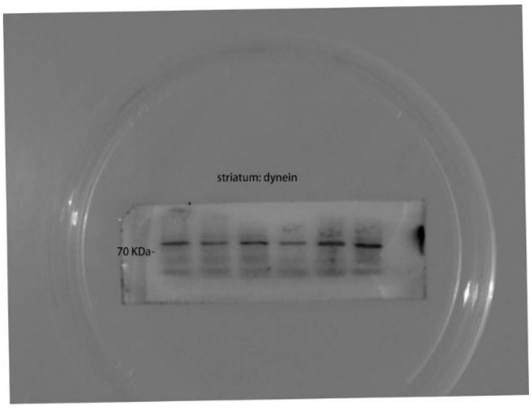

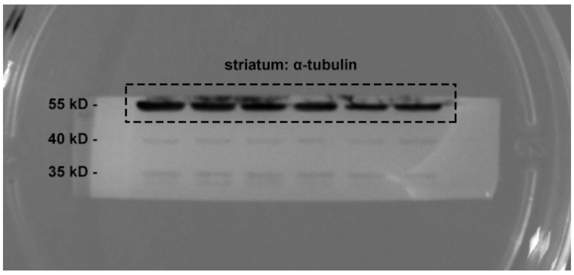

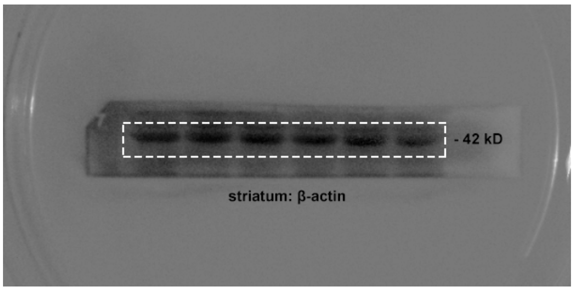

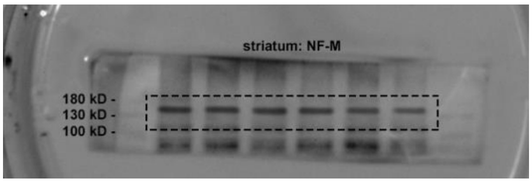

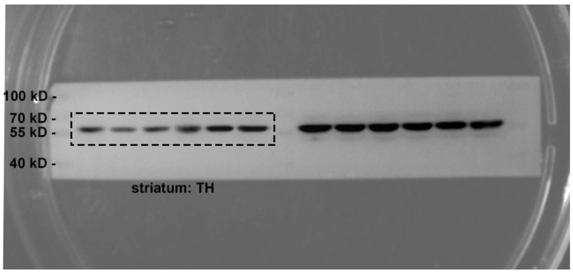

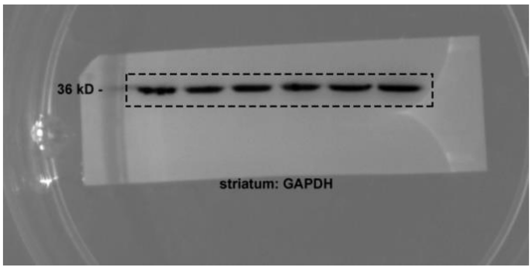

2.4.2 VBM (SN):

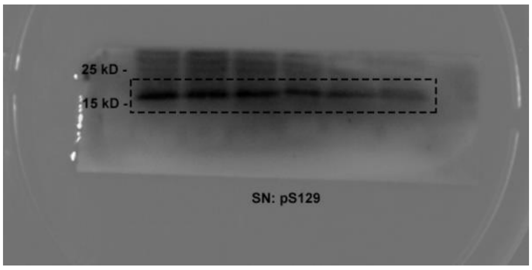
25 kD -
15 kD -
SN: pS129

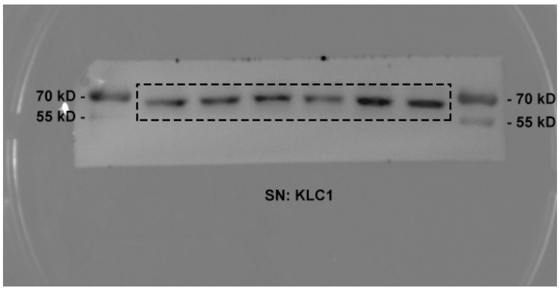
70 kD -
55 kD -
- 70 kD
- 55 kD
SN: KLC1

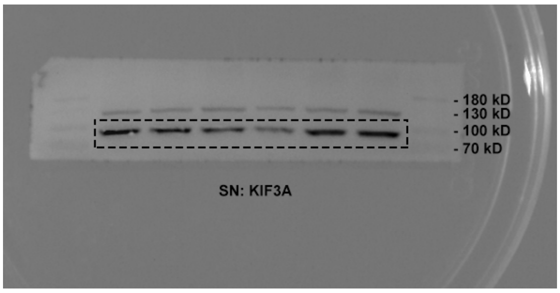
- 180 kD
- 130 kD
- 100 kD
- 70 kD
SN: KIF3A

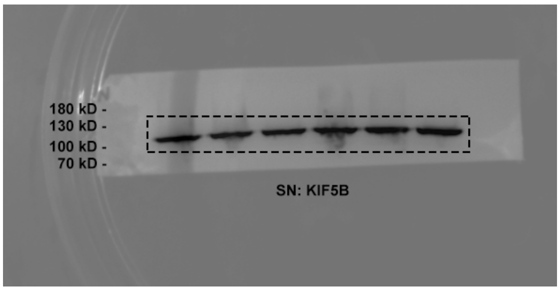
180 kD -
130 kD -
100 kD -
70 kD -
SN: KIF5B

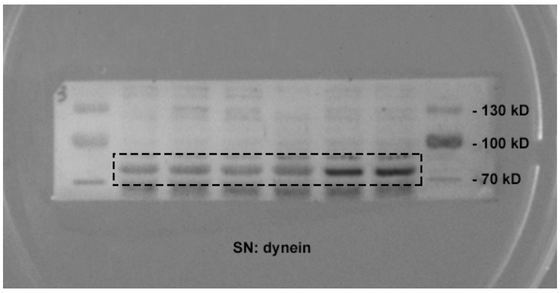
- 130 kD
- 100 kD
- 70 kD
SN: dynein

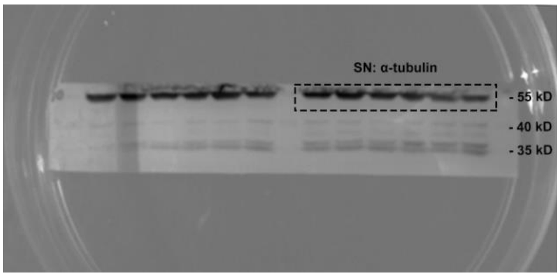
SN: α-tubulin
- 55 kD
- 40 kD
- 35 kD

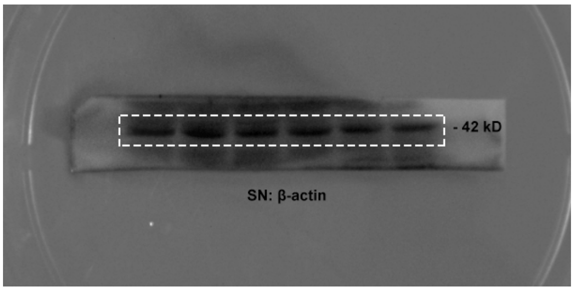
- 42 kD
SN: β-actin

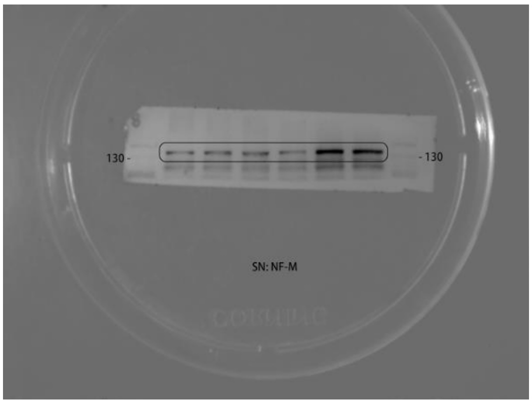
130 -
- 130
SN: NF-M

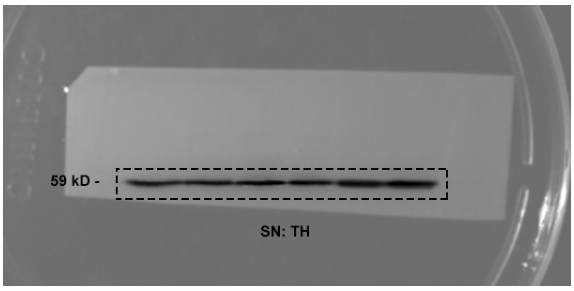
59 kD -
SN: TH

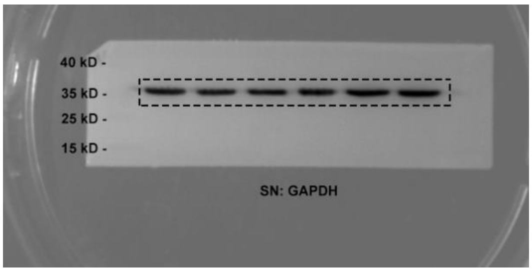
40 kD -
35 kD -
25 kD -
15 kD -
SN: GAPDH

2.5 Full unedited blots for supplemental Figure 4:

2.5.1 Striatum:

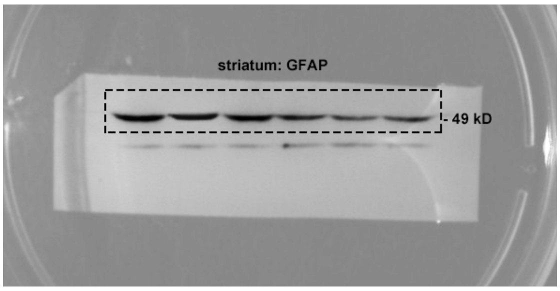

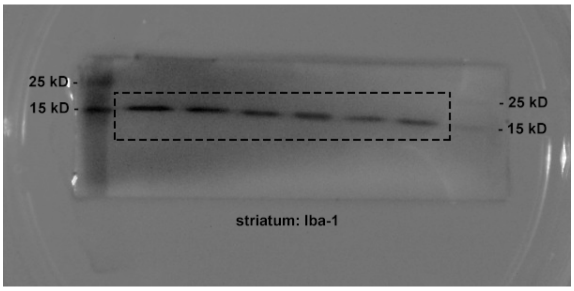

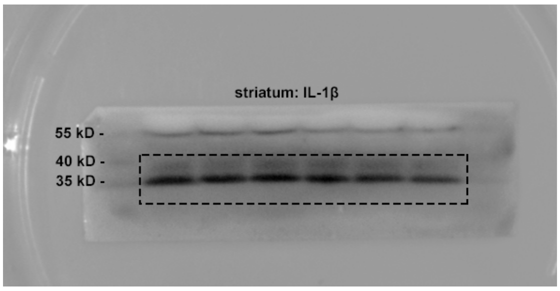

2.5.2 VBM (SN):

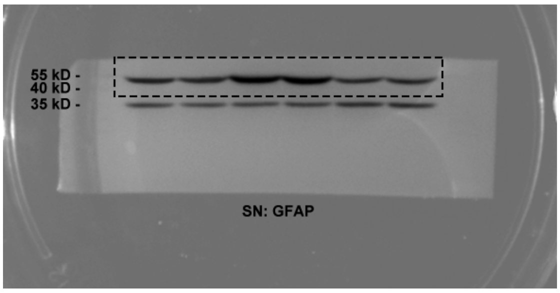

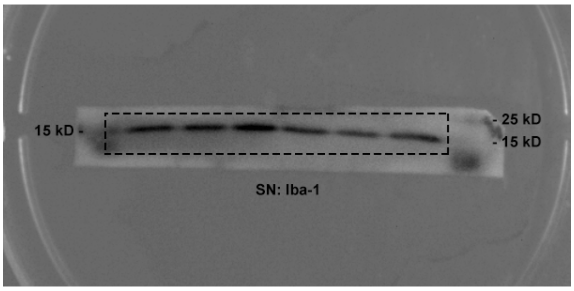

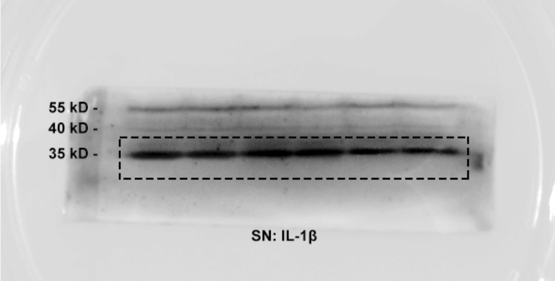

2.6 Other unedited blots not shown in figures:

2.6.1 Striatum: from top to bottom, 1 month, 2 months, 3 months and 6 months post injection

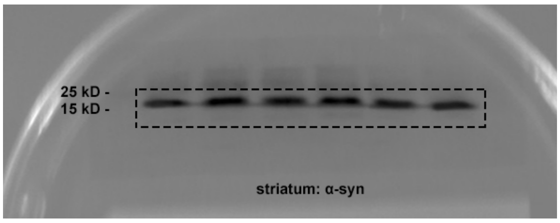

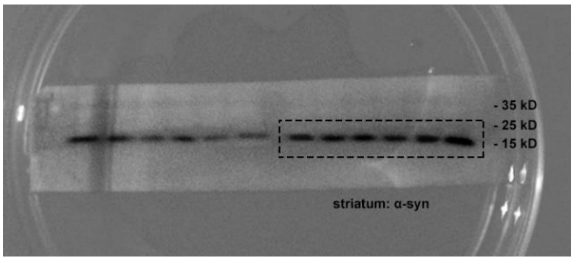

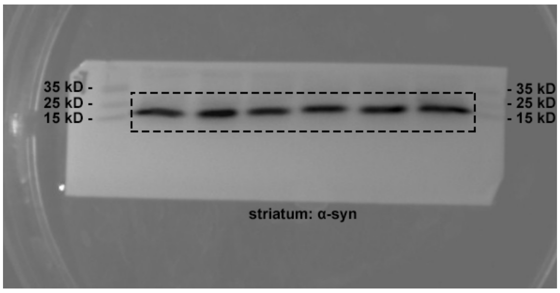

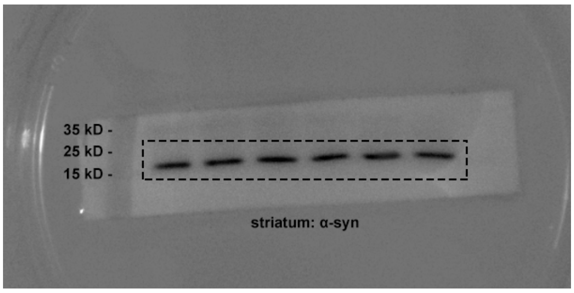

2.6.2 VBM (SN): from top to bottom, 1 month, 2 months, 3 months and 6 months post injection

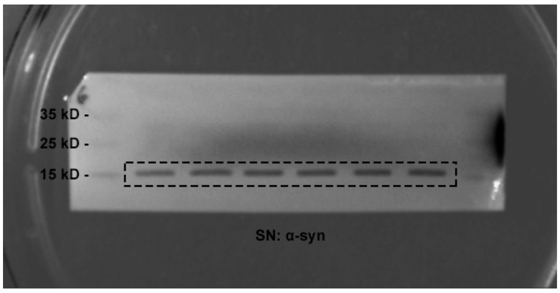

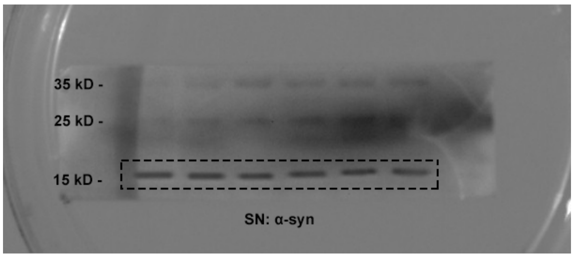

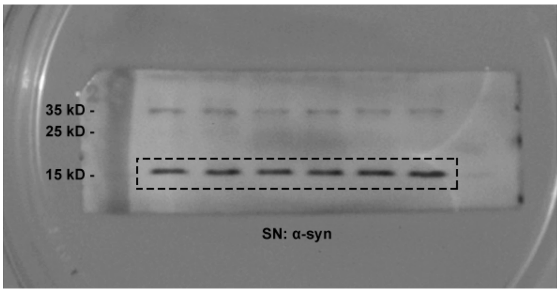

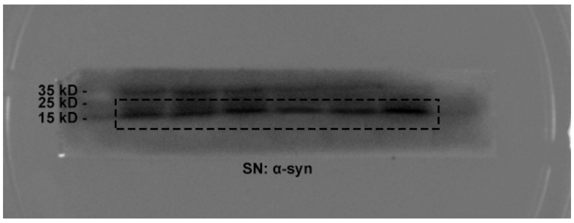

Supplement: Supplementary file 1 [file Data_Sheet_1.pdf]
